# Impacts on Sedimentary Microbial Communities Related to Temporal Changes in Trace Metal Concentrations

**DOI:** 10.1111/gbi.70027

**Published:** 2025-07-08

**Authors:** Christopher K. Jones, Jessica M. Labonté, Lauren A. Haygood, Marta E. Torres, Gerhard Bohrmann, Timothy W. Lyons, Natascha Riedinger

**Affiliations:** ^1^ Department of Earth and Planetary Sciences University of California, Riverside Riverside California USA; ^2^ Department of Marine Biology Texas A&M University at Galveston Galveston Texas USA; ^3^ Boone Pickens School of Geology Oklahoma State University Stillwater Oklahoma USA; ^4^ College of Earth, Ocean, and Atmospheric Sciences Oregon State University Corvallis Oregon USA; ^5^ MARUM‐Center for Marine Environmental Sciences and Faculty of Geosciences University of Bremen Bremen Germany

**Keywords:** diagenesis, manganese (oxyhydr)oxides, microbial communities, trace metals

## Abstract

Microbial processes in marine sediments drive changes in redox conditions, ultimately controlling the cycling of elements between the dissolved and solid phases. The microbial community driving these cycles depends on trace metals, but it can also be inhibited at elevated metal concentrations. During diagenesis, many trace elements are released from iron (Fe) and manganese (Mn) (oxyhydr)oxides, potentially affecting microbial metabolisms. Here we present results from geochemical and microbiological analyses of samples collected during R/V Polarstern Expedition PS119 to the East Scotia Ridge. The sediments are dominantly diatomaceous ooze with high contents of reactive Fe and Mn (oxyhydr)oxides and increased trace metal contents from nearby hydrothermal vents. Two multi‐corer cores were sampled immediately after collection at five specific sediment depths (three splits each), sealed anaerobically in incubation bags, and analyzed in 4‐month intervals post collection for major, minor, and trace metals and 16S rRNA gene sequencing. By isolating the sediment from overlying seawater during the incubation process, we simulated the in situ diagenetic processes of Fe and Mn oxide reduction. Our data show that Mn and trace metals, especially Mo, Ni, Tl, and Cu, are mobilized during early diagenesis. Analysis of 16S rRNA genes revealed shifts in the microbial community from Nitrososphaera and Nanoarchaeia to Bacteroidia and Bacilli alongside a marked decrease in richness, Pielou's evenness, and Shannon alpha diversity during the eight‐month incubations. We statistically correlate the microbial community shift with the changes in porewater trace metal concentrations, revealing that Mn, Co, Ag, and Tl are driving the microbial compositions in these samples. In this organic matter limited but Fe and Mn (oxyhydr)oxide rich system, we simulate deeper diagenesis to peer into the role of changing Fe, Mn, and trace metal cycles and highlight the role of Fe and Mn (oxyhdyr)oxides as shuttles for trace metals to the deep biosphere. By identifying key metals that are diagenetically cycled and affect the in situ microbial community, we reveal feedbacks between metals and microbial communities that play important roles in biogeochemical cycles on Earth, provide insight into the origin and potential evolution of metabolic pathways in the deep biosphere, and offer clues that may aid in our understanding of Earth's history and potentially beyond.

## 1 Introduction

Microorganisms are pivotal in cycling organic matter in marine sediments and do so through a series of metabolic pathways known as the redox cascade, which is defined by the preferential use of electron acceptors starting with oxygen and progressing to nitrate (NO_3_^−^), iron (Fe) and manganese (Mn) (oxyhydr)oxide phases, sulfate (SO_4_^2−^), and even uranium (U) (Froelich et al. 1979; Canfield 1993; Morford and Emerson 1999; Canfield 2006). However, in some systems, redox zonations exhibit non‐traditional pathways such as systems where electron acceptors are supplied by upward diffusion of brines and seawater from basaltic crust and by release of Fe and Mn from recalcitrant minerals that affect organic carbon cycling after other metabolic processes have become dominant in the sediments (Hensen et al. 2003; D'Hondt et al. 2004; Riedinger et al. 2005, 2014). Similar inverse redox zonations have also been observed in non‐marine environments with highly crystalline oxides playing a role in the cryptic sulfur cycle (Hansel et al. 2015).

Fe and Mn (oxyhydr)oxide phases are efficient and critical scavengers of trace metals from seawater, shuttling them from the water column to the sediments by adsorption onto the oxide surfaces (reviewed in Bruland et al. 2013). Iron and Mn (oxyhydr)oxide cycling is a major metabolic pathway for marine microorganisms (Froelich et al. 1979), and it is especially important in the sediments analyzed in this study. As Fe and Mn (oxyhydr)oxides are reduced during sediment diagenesis, dissolved Fe and Mn are released into the porewater (Froelich et al. 1979), and the trace metals that were adsorbed to these phases are also released back into the porewater where they can potentially diffuse out of the sediment or react with authigenic or residual minerals (e.g., McManus et al. 2006, and references therein). Once SO_4_^2−^ is reduced and hydrogen sulfide has formed, sulfidation of the released metals or co‐precipitation with sulfide minerals such as pyrite can immobilize specific trace metals (e.g., Emerson and Huested 1991). These sulfide minerals often remain stable, thus ultimately trapping nickel (Ni), molybdenum (Mo), cadmium (Cd), U, and other elements in the sediment (Morford and Emerson 1999; Canfield and Thamdrup 2009). The reduction of these oxides and formation of authigenic minerals is a complex process with many back reactions and alternate pathways.

Adsorption onto the surface of Fe and Mn (oxyhydr)oxide grains plays a major role in early diagenetic cycling and recycling of elements between the porewater and solid phase (reviewed in Tribovillard et al. 2006; Bennett and Canfield 2020). For example, Ni, Cu, Co, and Pb are primarily associated as cations (X^2+^), while other metals are associated as ionic species such as Mo (MoO_4_^2−^), V (VO^2+^), and Cr (Cr(OH)_2_^+^) (Elderfield 1970; Bruland 1983; Emerson et al. 1983; Calvert and Pedersen 1993). In addition, not all trace metals adsorb onto both Fe and Mn (oxyhydr)oxides: V and Cu readily adsorb onto both Fe and Mn (oxyhydr)oxides, but Mo and Ni are associated mainly with Mn(oxyhydr)oxides and show less affinity to Fe(oxyhydr)oxides (Tribovillard et al. 2006 *and references therein*; Goldberg et al. 2009). This sets the stage for the sequential release of adsorbed trace metals from Mn (oxyhydr)oxides first, as that is more energetically favorable, followed by Fe (oxyhydr)oxide associated metals later (Froelich et al. 1979; Morford et al. 2005). Upon reduction, both adsorbed trace metals and Fe and Mn are released into the porewaters where they can accumulate and undergo further reactions or be transported either by diffusion or advective fluid flow. For Fe, the primary sink is in authigenic sulfide phases, though Mn typically diffuses upwards or can precipitate as MnCO_3_ (Pedersen and Price 1982; Canfield, Thamdrup, and Hansen 1993).

In brief, if dissolved metals do not diffuse out of the sediments, many will eventually react with dissolved sulfide in the porewaters that is formed from bacterial sulfate reduction (Calvert and Pedersen 1993). As reviewed in Jørgensen et al. (2019), porewater Fe, as Fe(II), can react with sulfide (H_2_S + HS^−^ + S^2−^) and form authigenic sulfide minerals, though these reactions are not linear and have many back‐reactions, particularly oxidation by residual Fe(oxyhydr)oxides in the sediments. Some metals, such as Co, Ni, Cu, and Cd, can form either individual sulfide phases (e.g., CoS) or incorporate as solid solutions in pyrite (FeS_2_). Molybdenum, while still incorporated into sulfides, has a complex pathway involving the conversion of MoO_4_^2−^ to thiomolybdate and potentially requiring longer timescales (i.e., more than seasonal/intermittent euxinia) and more consistent sulfide concentrations (Erickson and Helz 2000; Tribovillard et al. 2006). Thiolation is perhaps a critical process in the activation of Mo prior to consumption in the solid phase, involving the replacement of an oxygen with a sulfur (MoO_4_^2−^to MoO_x_S_4‐x_) (Adelson et al. 2001). Other important processes are the formation of Fe‐Mo‐S clusters, which can be in solid solution with Fe‐sulfides or retained on their surfaces (Helz et al. 1996; Bostick et al. 2003) and scavenging of Mo by organic material (Bennett and Canfield 2020).

Early experiments in trace metal speciation and extraction identified Fe and Mn oxides, as well as organic matter, effectively scavenge metals (Tessier et al. 1979), which may induce competition for adsorption sites. This has been described for MoO_4_^2−^ which competes with other anions including sulfate, phosphate, and selenate for adsorption onto Mn oxides such as birnessite (Matern and Mansfeldt 2015). There is similar competition during Mo co‐precipitation with iron sulfides or on other solid phases in anoxic waters (Bertine 1972). Notably, adsorption properties and behaviors depend on a variety of factors including time, pH, and the surface area available, with Mn oxides typically having a higher specific surface area than aluminum or iron oxides (Matern and Mansfeldt 2015). Competition for adsorption sites may play a role in re‐adsorption of metals in closed systems such as the incubation experiments designed for this experiment.

The redox cascade in marine sediments is driven on a base level by the amount and quality of organic matter for microorganisms to use in metabolic processes (e.g., Henrichs and Reeburgh 1987; Henrichs 1992; Rullkötter 2006, and references therein). In addition to being the driver of early diagenetic processes, organic matter can act as a carrier for some trace metals, such as Cd, Ni, and silver (Ag), to the sediments (Huerta‐Diaz and Morse 1990, 1992; Morse and Luther 1999; Morford and Emerson 1999; Morford et al. 2001; Wagner et al. 2013). Higher amounts of labile organic matter can fuel rapid and intense diagenetic processes resulting in contracted redox zones as the individual terminal electron acceptors are used quickly (Canfield 1993). In contrast, sediments that are low in organic matter may instead have protracted redox zones, permitting sustained solubility of redox‐sensitive elements that might not be reflected in sediment cores at sites with higher organic carbon accumulation rates.

Microbial metabolic processes can cause the alteration of primary trace metal signals during burial in marine sediment, thus complicating interpretations of the original depositional conditions (Froelich et al. 1979). Microorganisms are known to use many trace metals (e.g., Mo, Ni, Cu, and V) as nutrients in metabolic processes and as metal cofactors for the enzyme nitrogenase (e.g., Kuypers et al. 2018; Lyons et al. 2024). However, metals such as Mo can also inhibit microbial growth (Biswas et al. 2009). For example, at concentrations of 2 mM, molybdate (MoO_4_^2−^) can almost entirely inhibit SO_4_^2−^ reduction and thus restrict the growth of SO_4_^2−^ reducing bacteria (Biswas et al. 2009). Even at lower concentrations of MoO_4_^2−^, down to 0.1 mM, there can be up to 40% inhibition of SO_4_^2−^ reduction (Biswas et al. 2009). Similarly to Mo and Cu, some microorganisms can incorporate and tolerate thallium (Tl) concentrations above open ocean concentrations of 10–20 ng L^−1^, often leading to an accumulation within the cell, though concentrations of ~50 nM begin to inhibit growth of organisms such as the chlorophyte *Micromonas pusilla* (Zhang and Rickaby 2020). The cyanobacteria *Synechococcus*, however, can tolerate up to 1 mg L^−1^ and did not accumulate Tl, demonstrating that responses to metal concentrations differ among the microbial community (Zhang and Rickaby 2020).

As metals are released to the porewater, there is the possibility of both inhibition, described above, and stimulation of a variety of microbial metabolic pathways, creating openings for change in the in situ microbial community. For example, the microbial methane and nitrogen cycles require Fe, Ni, Cu, Mo, and other metals as metal co‐factors, but these metals often have limited bioavailability (Glass and Orphan 2012). In laboratory and lake systems, N_2_O accumulation has been observed and attributed to Cu limitation (Glass and Orphan 2012), implying that, if metals such as Cu were released during diagenesis, they could stimulate various metabolic pathways. On the early Earth, this has been discussed for Ni in particular because of its role in methanogenesis (Konhauser et al. 2015). Archean iron formations record a decrease in Ni/Fe ratios, implying reduced Ni availability and potential limitations for methanogenesis, opening the doorway to the Great Oxygenation Event 2.5‐2.0 billion years ago (Konhauser et al. 2015). However, one of the uncertainties in this study of Archean iron formations is the role of diagenesis, and we investigate relevant diagenetic and microbial community changes through this incubation experiment.

Total metal content and its effect on the microbial community have previously been investigated, though these studies focused on metal‐rich settings such as acid mine drainage sites or lake systems with metal concentration gradients (e.g., Gough and Stahl 2011; Kwon et al. 2015). For example, heavy metals such as Cu, zinc (Zn), or lead (Pb) show positive correlations between metal concentration and some members of the microbial communities, such as Bacteroidia (Li et al. 2020).

Porewater and solid phase metal content of marine sediments has been studied at many sites with a strong focus on continental margins and near anthropogenic activity, which can supply macronutrients and trace metal micronutrients to the water column and sediments (Hurst and Bruland 2008; Chen et al. 2022). Furthermore, some studies have investigated and characterized the microbial communities present in high metal content sediments, particularly in human‐impacted and polluted sites such as acid mine drainage or sewage systems (Sullivan and Yelton 1988; Lion et al. 1988; Di Cesare et al. 2020). However, studies showing the response through time of the in situ microbial community to a sustained release of metals during early diagenesis in marine sediment are very limited. To investigate this further, we designed an incubation experiment that combines both inorganic geochemical analyses with microbiological characterization to assess the processes and effects of diagenesis on the in situ microbial community. The incubations were kept as closed systems to isolate the role of hydrothermally sourced Fe and Mn (oxyhydr)oxides, and the metals adsorbed onto or included within them, during diagenesis and the consequences of metal cycling on microorganisms.

Here, we present combined trace metal analyses with the identification of the microbial community composition during diagenesis in the uppermost sediments of the Scotia Sea to investigate the interlink between microbial communities and trace metal release into and/or sequestration out of the porewater by simulating diagenetic cycling in a closed system. This study illuminates the role of trace metals in microbial ecology in modern marine sediments and the potential impact on mineralization and metabolic rates.

## 2 Materials and Methods

### 2.1 Study Area

The South Sandwich Island Arc formed by the subduction of the South American plate beneath the Sandwich micro‐plate, and they supply ash and lithogenic material through submarine landslides to the seafloor in the Scotia Sea (Cole et al. 2014; Leat et al. 2016). Additionally, the East Scotia Ridge has active, predominantly black smoker‐type hydrothermal vents which also supply material to the sediments (German et al. 2000; James et al. 2014). Sediments in this area are characterized by low terrestrial input, low amounts of organic matter, and are composed primarily of diatomaceous ooze, clays, and silts (Gersonde 1993; Moreton and Smellie 1998). Non‐biogenic detritus is mainly delivered from the South Sandwich Islands with additional contributions from the Antarctic Peninsula and Patagonia region of South America. The detrital contribution is dominated by basic and undifferentiated crustal material (Diekmann et al. 2000), and from hydrothermal discharge at the northern ridge segment, which is characterized by hot fluids (mainly > 300°C; James et al. 2014; Pereira et al. 2022). The nearby hydrothermal vent activity supplies high amounts of Fe and Mn (oxidized in seawater to highly reactive oxide‐minerals) as well as trace and rare earth metals (Crocket 1990; Sander and Koschinsky 2011; Yücel et al. 2011; Hawkes et al. 2014), which make these sediments prime samples for investigating diagenesis and sediment trace metal cycling. The very low rates of organic matter accumulation (e.g., Harloff and Mackensen 1997) lead to expanded redox zones, allowing for the high‐resolution sampling of diagenetic processes that may be missed in other sampling locations that undergo more rapid organic matter remineralization.

### 2.2 Sampling and Incubation Procedure

**FIGURE 1:**
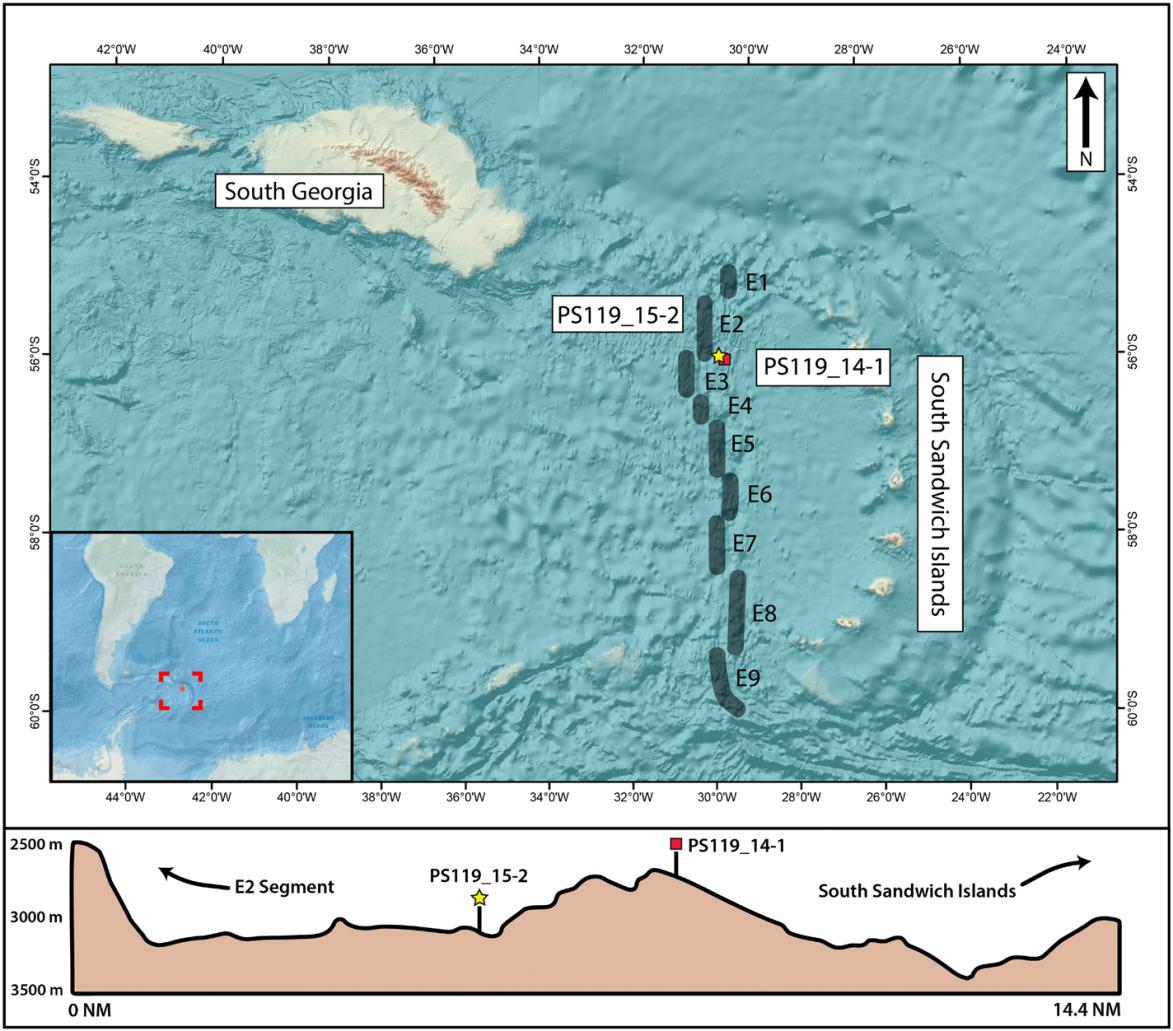
Location of multi‐corer cores PS119_14‐1 and PS119_15‐2 collected from the East Scotia Ridge (ESR) during R/V Polarstern Expedition PS119. The yellow star indicates the location of Site PS119_15‐2, and the red, solid box indicates the location of Site PS119_14_1. The nine segments of the East Scotia Ridge hydrothermal vent system are labeled E1–E9 and marked by gray lines based on segment positions in German et al. (2000). Study area relative to South America, Africa, and Antarctica is denoted by the red box and red star in the inset map found at the lower left. Bottom panel depicts the sampling locations of both cores and the topography, based on echosounder data, along a 14.4 nautical mile, NW‐SE cross section. Black, leftward arrow indicates the direction towards the E2 Segment of the ESR, and the black, rightward arrow indicates the direction towards the South Sandwich Island Arc.

**FIGURE 2:**
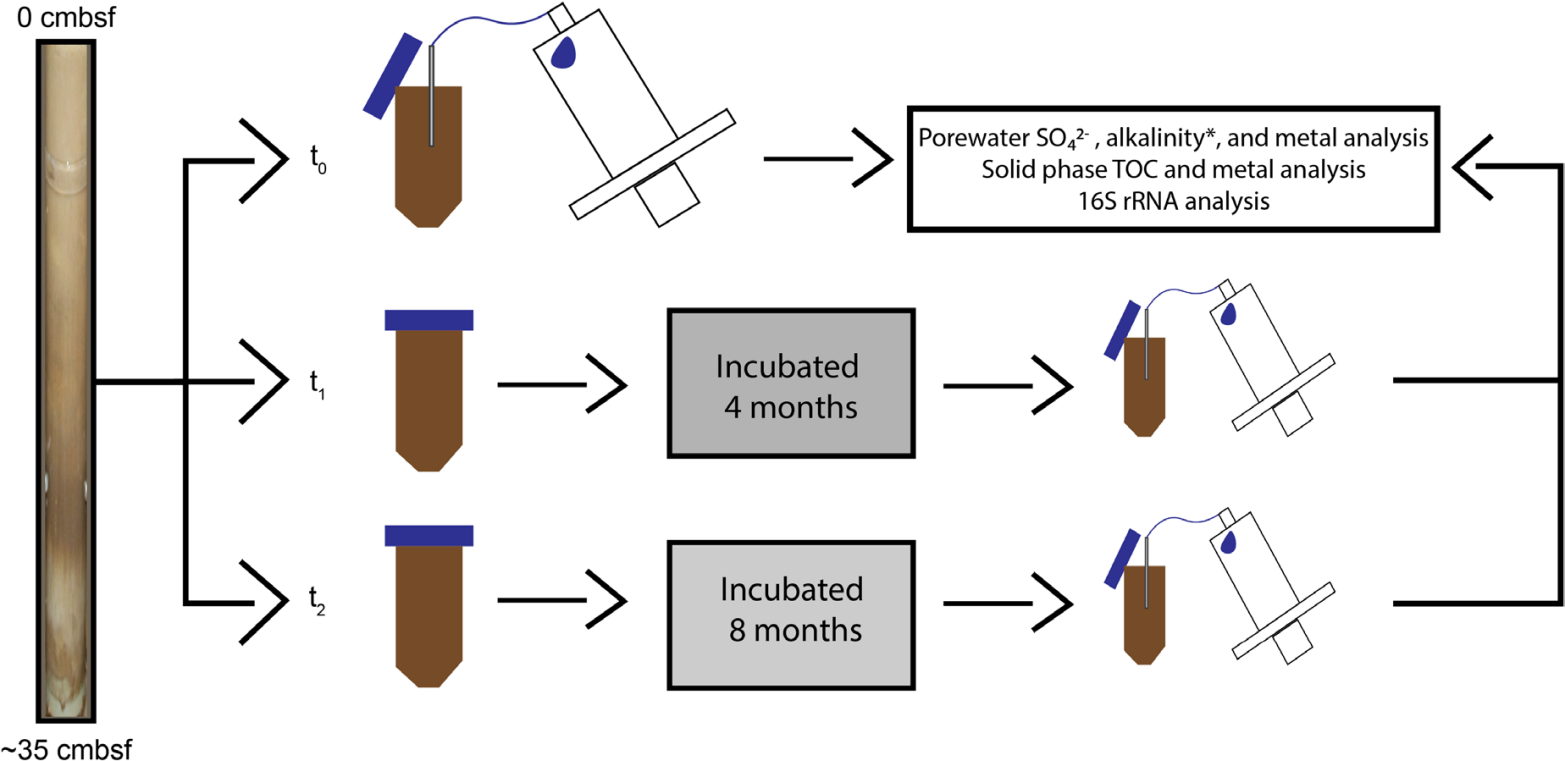
Schematic of the sampling, incubation, and analytical procedures. Samples were recovered with a multi‐corer. Recovered cores were ~35 cm in length spanning from the sediment–water interface at 0 cm below seafloor (cmbsf) to 35 cmbsf. Cores were then sectioned using sterile tools into 50 mL centrifuge tubes, sealed in nitrogen flushed aluminum foil bags, and stored at 4°C for an incubation period (directly after sampling, *t*_0_, 4 months, *t*_1_, and 8 months, *t*_2_). Following incubation, porewater was extracted using Rhizons for analysis of sulfate (SO_4_^2−^) alkalinity (only *t*_0_ samples*), and metal concentrations. After porewater extraction, a sediment split was removed for inorganic geochemical analysis of total organic carbon (TOC) and metal content, and the remaining sediment was preserved for 16S rRNA analysis.

Samples were collected in 2019 during R/V Polarstern Expedition PS119 to the Scotia Sea (Figure 1). Two, ~35 cm long multi‐corer cores (MUC) retrieved within 20 km east of an active hydrothermal vent on ridge segment E2 were selected for subsampling: MUC PS119_14–1, recovered from a local topographic high of 2763 m water depth (−56.12842, −30.0696), and MUC PS119_15‐2, from 3034 m water depth (−56.10997, −30.15214) in the nearby topographic low (Bohrmann 2019). The sampling protocol is illustrated in Figure 2. Sediments were transferred, using sterile tools, to 50 mL centrifuge tubes and immediately brought to a cold room at 4°C. Porewater from the first split (*t*_0_) was extracted on board using Rhizons, with 13 samples collected from PS119_14‐1 and 14 samples from PS119_15‐2. After porewater extraction, the remaining sediment sample was frozen at −20°C for trace metal analysis on shore. Subsamples for microbiological analysis for t_0_ were collected at intervals between the porewater sampling depth, and the samples were handled with sterilized equipment and in a manner to avoid contamination and stored at −80°C. For sets *t*_1_ and *t*_2_ (Figure 2), the incubated sediments were used for both inorganic geochemistry and microbiological analysis. The incubation was intended to simulate diagenesis to investigate the role that cycled metals affect the in situ microbial community by combining inorganic geochemical analyses with microbiological analyses on the same samples. For *t*_1_ and *t*_2_, only five depths were sampled by transferring the sediment to sterile centrifuge tubes, which were sealed in argon‐flushed, heat‐sealed aluminum bags, and stored at 4°C to simulate the seafloor environment and diagenetic cycling in a closed system. Splits *t*_1_ and *t*_2_ were opened and had porewater extracted after 4 months (*t*_1_) and 8 months (*t*_2_), respectively. They were then analyzed for porewater SO_4_^2−^ and trace metal concentrations. Following each porewater extraction, a sediment split was taken using sterile equipment, dried at 38°C for 48 h, homogenized with an agate mortar and pestle, and transferred to storage vials for future solid phase analysis of total organic carbon and trace metal content. The remaining sediment was frozen at −80°C before being processed for microbiological analysis.

### 2.3 Porewater

Porewater was extracted from the sediment samples via Rhizons and metal‐clean syringes (Seeberg‐Elverfeldt et al. 2005). The first 1 mL of porewater was discarded to prevent contamination and dilution from residual fluid in the Rhizon filter. Subsamples were taken on the first sample set (*t*_0_) for alkalinity and major element analyses and all following sets for trace element analyses. For element analyses, a split of the extracted porewater was immediately acidified with ~10 μL concentrated trace metal grade HNO_3_ per mL porewater.

Alkalinity was measured onboard for all *t*_0_ samples, but alkalinity was not measured for incubated samples. Alkalinity was determined on a 1 mL aliquot of sample by titration with 10 or 50 mM HCl. The pH measurements were performed using a Mettler Toledo micro‐electrode. Samples were titrated to a pH of approximately 3.9 with a digital burette, and both titration volume and final pH were recorded. The alkalinity was calculated using an equation modified from Grasshoff et al. (1999).

Subsamples for SO_4_^2−^ analyses were diluted 25‐fold with 2% trace metal grade HNO_3_, and concentrations were determined by inductively coupled plasma‐optical emission spectroscopy (ICP‐OES) on a Thermo Scientific iCAP Pro at Oklahoma State University, with a general analytical error < 3%. For trace metal analyses, porewater aliquots were diluted 25‐fold with 2% trace metal grade HNO_3_ and measured using ICP‐mass spectrometry (MS) on a Thermo Fisher Scientific iCAP Qc MS. Standards were prepared from stock solutions and trace metal grade NaCl to match the salt matrix present in seawater. National Institutes of Standards and Technology (NIST) Standard Reference Material (SRM) 1643f was included for quality control, and measured values are within 9% of published SRM 1643f values.

### 2.4 Solid Phase

Total organic carbon (TOC) was analyzed using approximately 40 mg of powdered rock samples that were placed in silver capsules in a 24‐well plate. Samples were acidified to remove any inorganic carbon present and dried in an oven at 60°C for 3 days. Approximately 1 mg of vanadium pentoxide was added to each sample and wrapped in a silver capsule that was subsequently wrapped in a tin capsule. Samples were analyzed for TOC using a Costech ECS 4010 elemental combustion system. For each set of analyses, a set of standards, blanks, and replicates was prepared. The blanks included empty tin and silver capsules, and Acetanilide was used as a concentration standard for TOC, which is reported in units of weight percent (wt.%). Sample analysis error of replicate samples was better than 14%.

Between 80 and 120 mg of dried and homogenized sediment was weighed out into clean polytetrafluoroethylene (PTFE) vials. Total digest was carried out in a PicoTrace temperature and pressure control digest system using three supra‐pure acids: nitric (HNO_3_), perchloric (HClO_4_), and hydrofluoric (HF). The remaining residue after evaporation was weighed and dissolved in 10 mL 5% HNO_3_. Several samples were selected as duplicates and digested multiple times to check data quality along with a NIST Standard Reference Material (SRM) 2702 for modern marine sediments and blanks (see also Abshire et al. 2022). Solid phase digests were run for trace metal content on an Agilent 7900 ICP‐MS at the University of California, Riverside. Measured values above detection limits are within 12% of published SRM 2702 reference values.

### 2.5 Community Analysis Using 16S rRNA Gene Sequencing

Total DNA was extracted from 0.5 g of frozen samples of core PS119_14‐1 (from sets A and C) using the DNeasy PowerSoil kit (Qiagen, USA) and stored at −20°C until polymerase chain reaction (PCR) amplification. A negative control using 0.5 mL of 0.2 μm filtered, sterile MilliQ water used for extraction and PCR was also extracted to identify possible contamination from the ambient lab and kit reagents. PCR amplification, using Platinum Taq DNA Polymerase (ThermoFisher Scientific), was performed following the 16S rRNA gene Illumina amplicon protocol from the Earth Microbiome Project (Thompson et al. 2017). Each sample was amplified in triplicate 25 μL reactions with the following cycling parameters: 95°C for 3 min, 30 cycles of 95°C for 45 s, 50°C for 60 s, and 72°C for 90 s, and a final elongation step at 72°C for 10 min. The V4 hypervariable region of the 16S rRNA gene was amplified using the updated 515F‐806R primer pair (10 μM each) from Parada et al. (2015) modified to include Golay barcodes and adapters for Illumina MiSeq sequencing as detailed in Walters et al. (2016). Following amplification, the triplicate products were combined and run on a 1.5% agarose gel to assess amplification success and relative band intensity. Successful reactions were then purified with the Qiagen PCR Clean‐up kit (Qiagen, USA). The products were subsequently purified with Ampure XP beads (Beckman Coulter) to further remove primer dimers. Approximately 50 ng of each sample was pooled, and the purified products, along with aliquots of the sequencing primers, were sent to the Texas A&M Genomics and Bioinformatics Services (College Station, TX, USA) for MiSeq sequencing (v2 Nano chemistry, 2 × 250 bp).

The sequences were analyzed using DADA2 following the DADA2 Pipeline Tutorial v1.16 (Callahan et al. 2016), using the SILVA alignment v138 for taxonomic identification. Any ASV within the genus of the amplicon sequence variants (ASVs) that were found in the negative control at a relative abundance > 0.5% were removed for further analyses. The diversity indices were calculated using the diversity command in the *vegan* package (Oksanen et al. 2020) in R using the distribution table of all the ASVs identified.

### 2.6 Statistical Analyses

The non‐metric multidimensional analysis was performed using the *envfit* function (permutations = 9999) available in the *vegan* R package (Oksanen et al. 2015) and plotted with ggplot2 (Wickham 2016), using the taxa (class level) relative abundances and log‐transformed environmental variables (geochemical data). The correlations between the taxa and the metadata were identified with the *rcorr* function available in the *Hmisc* package 4.7 on the relative abundance of the ASVs against the raw metal concentrations. The cluster analysis was performed using the *hclust* function on a distance matrix of the raw metal concentrations.

## 3 Results

**FIGURE 3:**
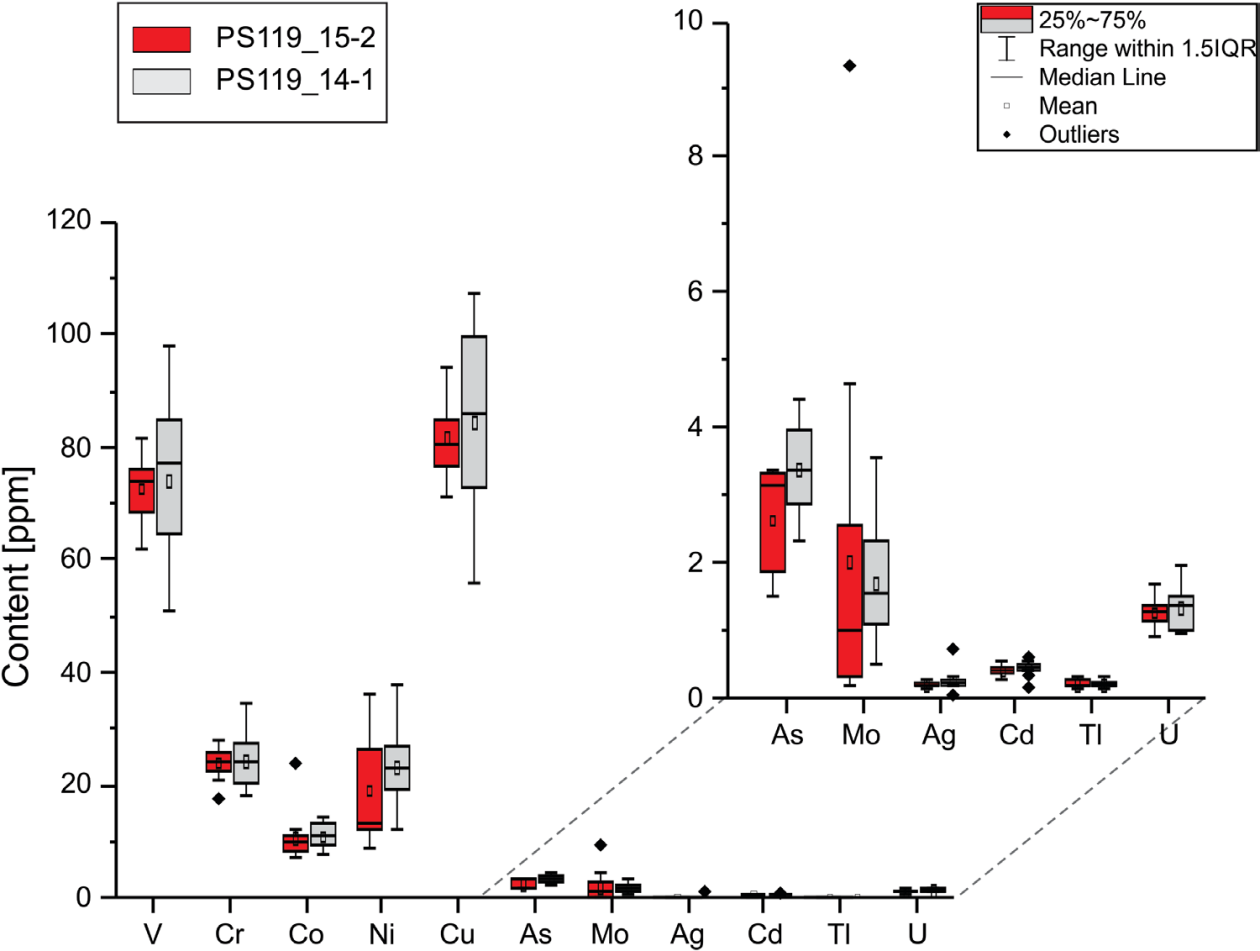
Solid phase metal content ranges at Sites PS119_14‐1 (gray) and PS119_15‐2 (red). The colored rectangles show 25%–27% of the occurrence with the horizontal line as the median line, the range within 1.5 interquartile range shown by the vertical line, rhombus indicates outliers, small squares depict the median value.

Both investigated sites, PS119_14‐1 and PS119_15‐2, are dominated by siliceous ooze, typical of Scotia Sea sediments (Moreton and Smellie 1998; Weber et al. 2021). Manganese and Fe contents are 0.14–0.31 wt.% Mn and 1.87–4.02 wt.% Fe at Site PS119_14‐1, and 0.04–0.52 wt.% Mn and 2.38 to 4.13 wt.% Fe at Site PS119_15‐2. Aluminum ranges between 2.56 and 5.62 wt.% and 3.10 wt.% and 5.65 wt.% at Sites PS119_14‐1 and PS119_15‐2, respectively. Trace metal contents are comparable in both cores (Figure 3), with V and Cu as the most abundant trace metals averaging ~80 ppm (Tables S3 and S4). For both V and Cu, the average and range are higher in sediments from PS119_14‐1 than in PS119_15‐2. Chromium, Co, and Ni contents range between 10 and 30 ppm, whereas As, Mo, Ag, Cd, Tl, and U contents are below 5 ppm. Of the 6 least abundant trace metals, Mo in PS119_15‐2 exhibits the widest content range in the solid phase.

### 3.1 Total Organic Carbon

**FIGURE 4:**
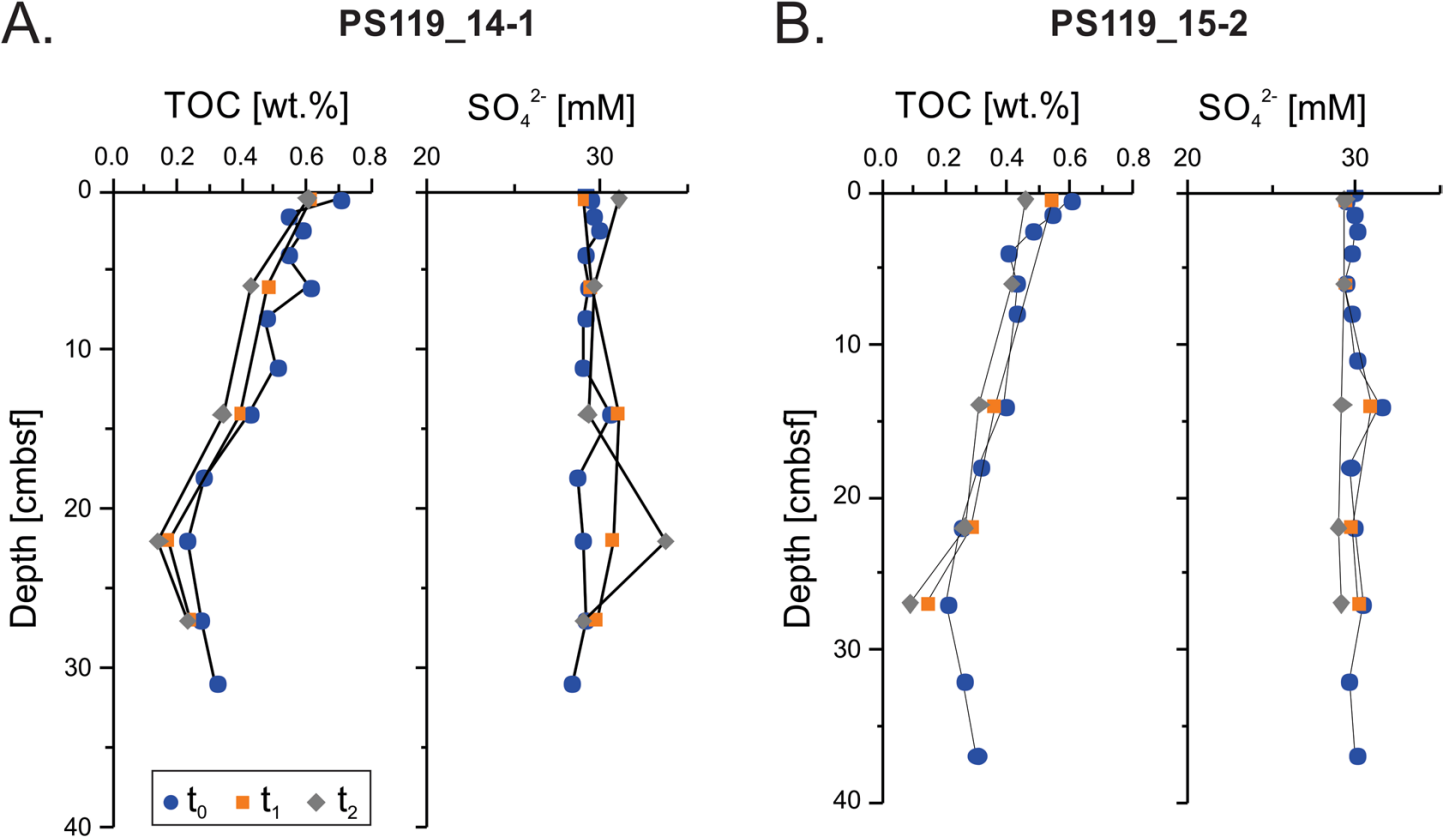
Total organic carbon (TOC) content and porewater sulfate (SO_4_^2−^) concentrations at Sites (A) PS119_14‐1 and (B) PS119_15‐2. All depths are in centimeters below seafloor (cmbsf). Samples were measured at 0 months (*t*_0_) (blue circles), 4 months (*t*_1_) (orange squares), and 8 months (*t*_2_) (gray diamonds) incubation.

Total organic carbon (TOC) contents are low at both sites, not exceeding 0.8 wt.%. There is an inflection point in both TOC profiles at 22 cm below seafloor (cmbsf) at Site PS119_14‐1 and 27 cmbsf at Site PS119_15‐2. Above these depths, TOC content decreases from a maximum at the top of the core to minima at 22 and 27 cmbsf, respectively. Below these depths, TOC values begin to increase with depth. During the incubations, the TOC content slightly decreases in the sediments from both sites (Figure 4). T‐tests were performed to evaluate statistical significance in differences in TOC content over time. The lowest *p*‐values of 0.014 and 0.097 were recorded when comparing *t*_0_ and *t*_2_ TOC contents of PS119_14‐1 and PS119_15‐2, respectively.

### 3.2 Porewater Constituents

Alkalinity values for *t*_0_ samples are low (between 2.50 and 2.98 mM) at both sites and show little variation with depth (Tables S5 and S6). The SO_4_^2−^ concentration profiles generally show very little variation at both sites throughout the cores in the initial sampling set (*t*_0_) (Figure 4). During the incubations SO_4_^2−^ concentrations increase slightly to values greater than bottom water concentrations of 29.1 and 30.0 mM at Sites PS119_14‐1 and PS119_15‐2, respectively, particularly at depths of ~22 cmbsf at Site PS119_14‐1 and ~12 cmbsf at Site PS119_15‐2 (Figure 4). All porewater Fe concentrations are at or below 1 μM at both sites in all samples, and there are no downcore trends in the dissolved Fe data (Tables S1 and S2).

**FIGURE 5:**
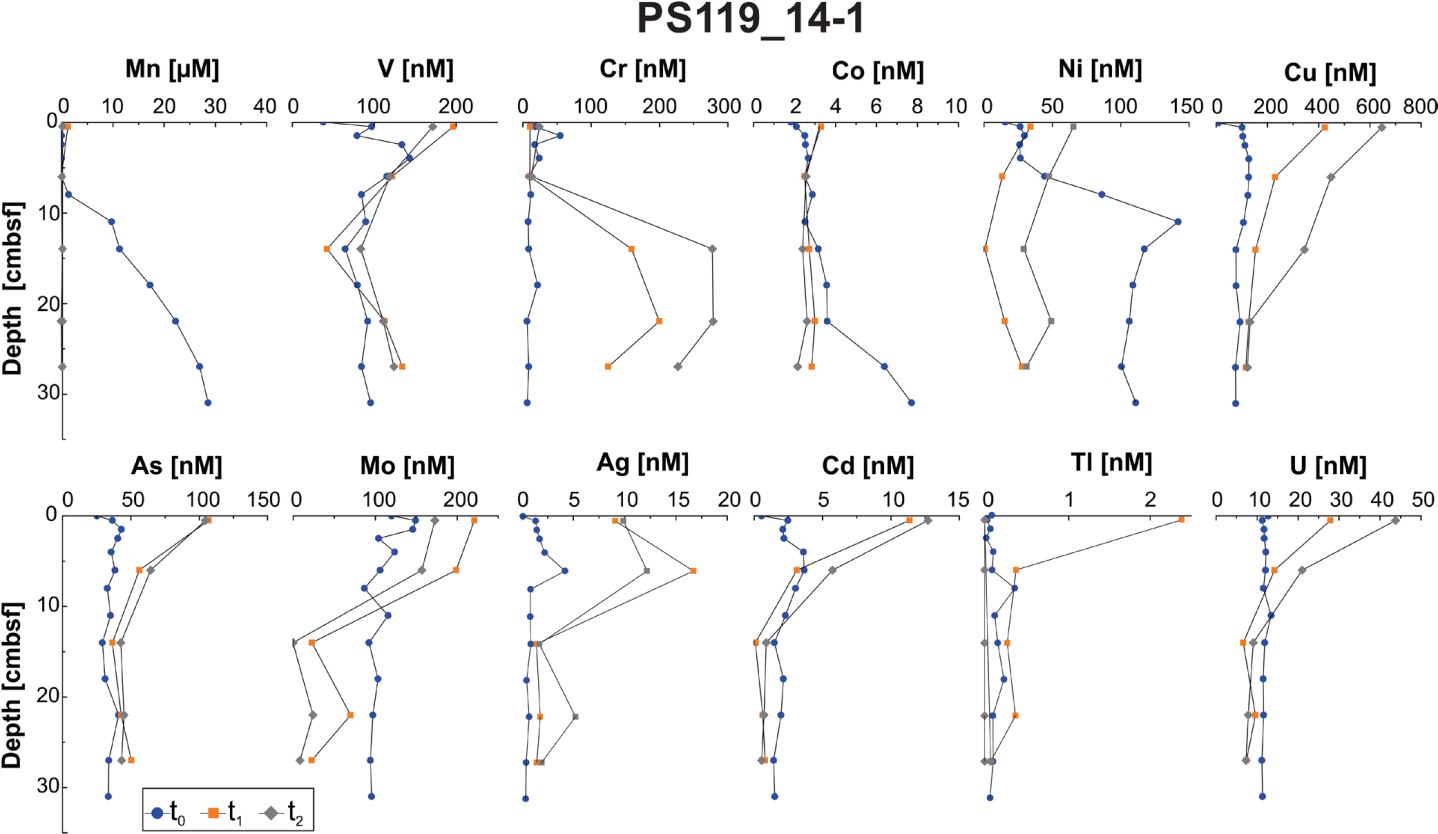
Porewater concentration profiles of manganese (Mn), vanadium (V), chromium (Cr), cobalt (Co), nickel (Ni), copper (Cu), arsenic (As), molybdenum (Mo), silver (Ag), cadmium (Cd), thallium (Tl), and uranium (U) at Site PS119_14‐1. All depths are in centimeters below seafloor (cmbsf). Samples were measured at 0 months (*t*_0_) (blue circle), 4 months (*t*_1_) (orange square), and 8 months (*t*_2_) (gray diamond) incubation.

**FIGURE 6:**
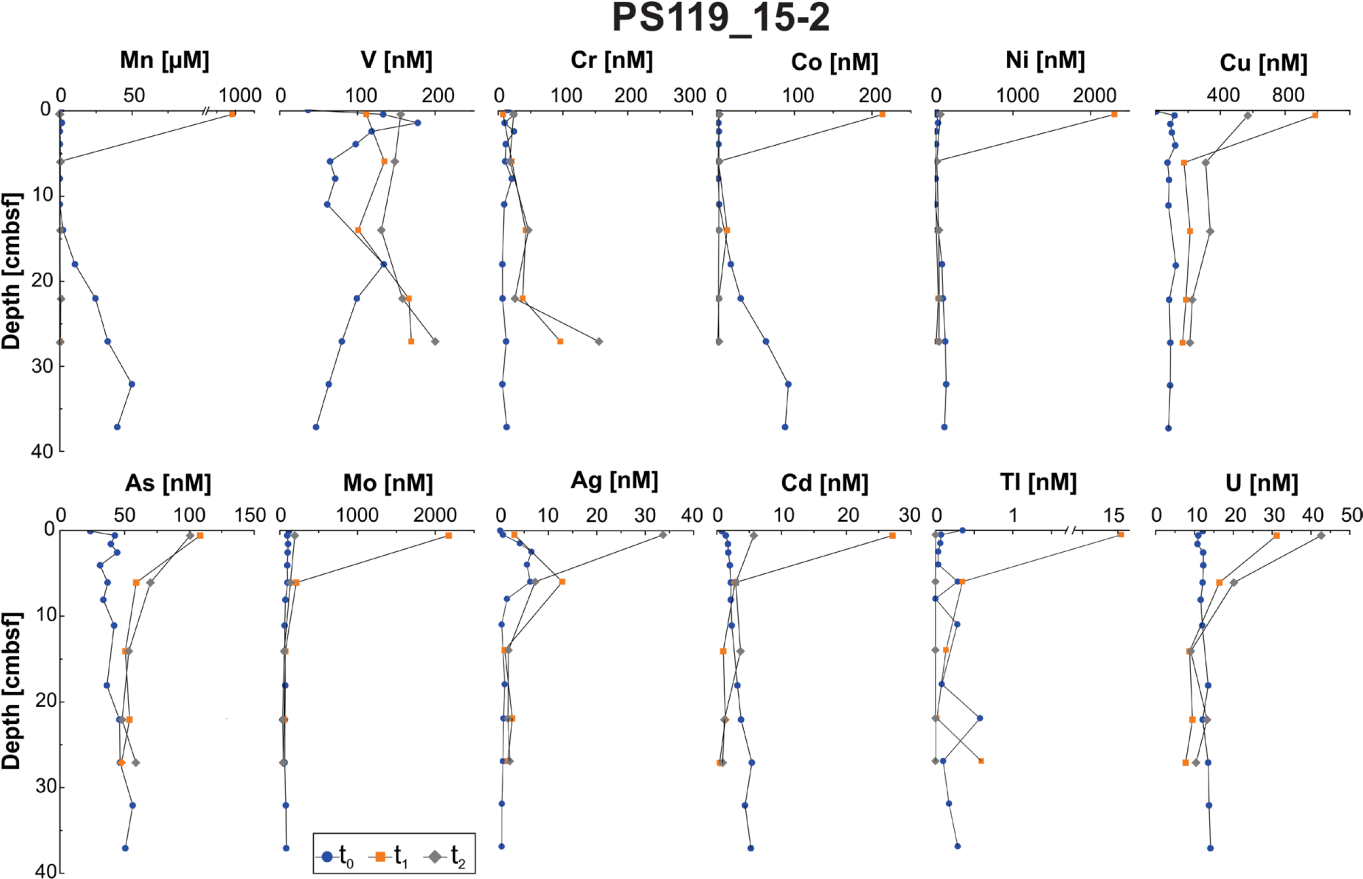
Porewater concentration profiles of manganese (Mn) (note the break in the *Y*‐scale), vanadium (V), chromium (Cr), cobalt (Co), nickel (Ni), copper (Cu), arsenic (As), molybdenum (Mo), silver (Ag), cadmium (Cd), thallium (Tl) (note the break in the *Y*‐scale), and uranium (U) at Site PS119_15‐2. All depths are in centimeters below seafloor (cmbsf). Samples were measured at 0 months (*t*_0_) (blue circle), 4 months (*t*_1_) (orange square), and 8 months (*t*_2_) (gray diamond) incubation.

The general trends in the porewater metal concentration profiles are comparable at both sites, but with notable differences discussed below. Most metals display higher concentrations in the uppermost, incubated samples followed by a down‐core decrease (Figures 5 and 6). The exception to this trend can be observed at t_0_ for Mn, Co, and Ni, as well as *t*_1_ and *t*_2_ Cr profiles of PS119_14‐1, and at t_0_ for Mn, Co, and Cd, as well as *t*_1_ and *t*_2_ Cr and V profiles of PS119_15‐2, where concentrations increase down‐core (Figures 5 and 6). Porewater trace metals from core PS119_14‐1 increase in concentration through the incubations, with the highest concentrations in all but Mn, Co, and Ni occurring in either *t*_1_ or *t*_2_ samples. The Ag concentration profiles indicate a release of Ag with the highest values around 5 cmbsf during the incubations (*t*_1_, *t*_2_). In the uppermost sample from core PS119_15‐2, Mn, Mo, Cd, Cu, Co, Ni, and Tl are elevated by an order of magnitude in the porewater in the first incubation sample (*t*_1_), while Ag is similarly elevated at *t*_2_ (Figure 6). Arsenic and U concentrations also increase in the uppermost sample from core PS119_15‐2, but not to such an extreme compared to the other metals (Figure 6). Chromium concentrations increase at both sites during the incubations, but the highest values are reached in deeper samples instead of the shallowest. In general, V concentrations increase during the incubations and increase below 15 cmbsf. Sediments from site PS119_14‐1 show a drastic increase in Tl to > 2 nM during *t*_1_ in the surface sediments. In core PS119_15‐2, Tl concentrations increase from 0.33 to 14.4 nM in the uppermost sample (Figure 6).

### 3.3 Microbial Community Analysis

To characterize the changes in the microbial community composition, we sequenced the 16S rRNA gene from the original core sample (*t*_0_) as well as at the end of the 8 months incubation period (*t*_2_). Each sample initially had between 9039 and 99,028 sequences (Table S7). After filtering the sequences with Ns, duplicates, and chimeras, each sample was left with between 4352 and 92,176 quality sequences. A total of 2783 unique sequences, or amplicon sequence variants (ASVs), were identified, with samples containing between 96 and 1027 ASVs. A total of five ASVs with > 0.5% of the sequences found in the negative control were identified. These ASVs, which were identified as belonging to the genera *Sphingomonas*, *Acidibacter*, and *Bradyrhizobium*, are known laboratory contaminants from DNA extraction and PCR reagents (Salter et al. 2014). Any sequences belonging to these genera were removed from further analyses.

**FIGURE 7:**
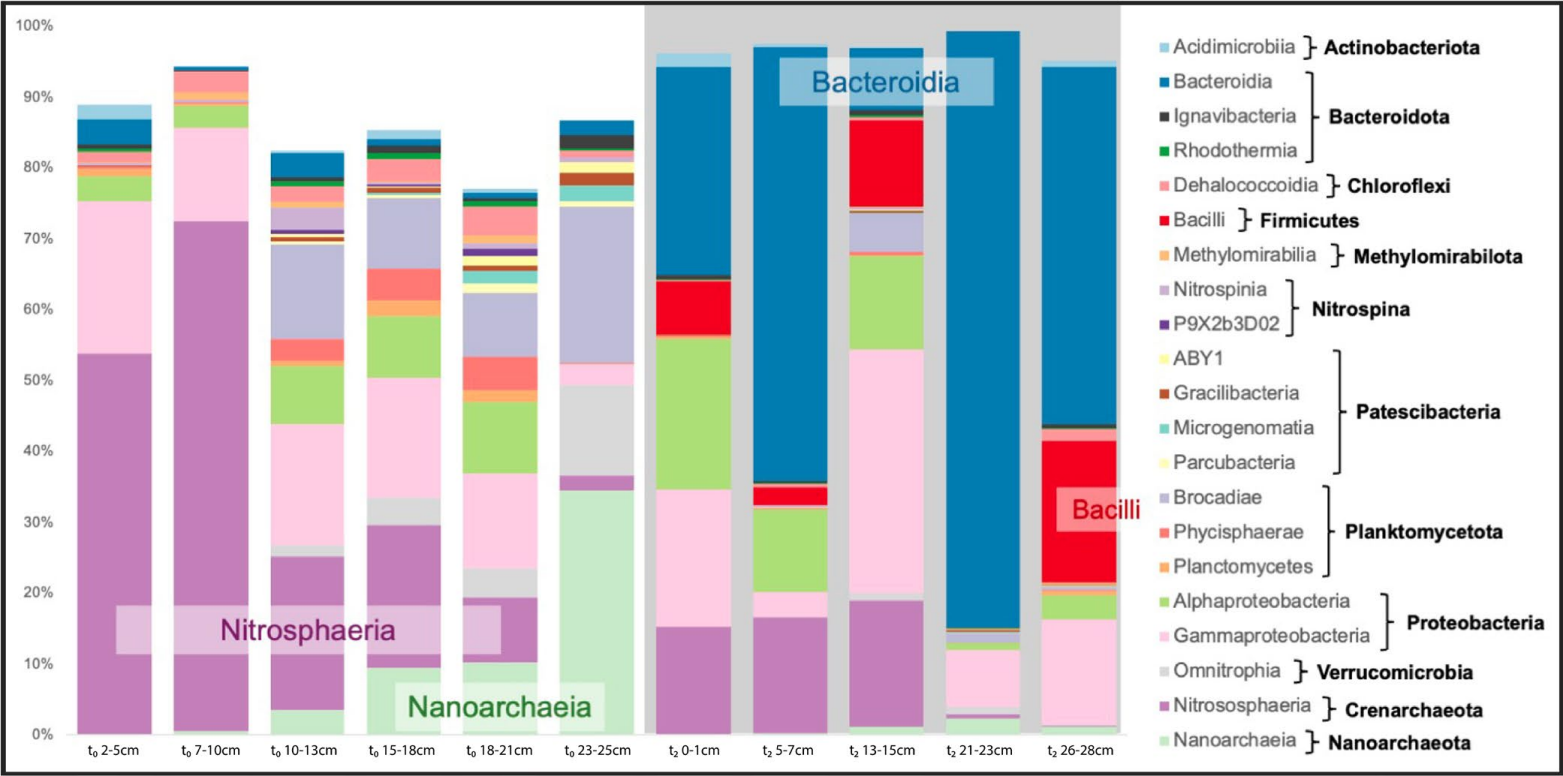
Taxonomic distribution at the class level of all the ASVs identified in the samples from PS119_14‐1. The left six columns labeled *t*_0_“sample” (e.g., *t*_0_ 2–5 cm) represent the 6 depths sampled for 16S rRNA gene sequencing from the initial, non‐incubated core. The right five columns, labeled *t*_2_“sample” (e.g., *t*_2_ 0–1 cm) represent the 5 corresponding depths incubated for 8 months and sampled for 16S rRNA gene sequencing.

The distribution of the ASVs was greatly impacted during the incubation simulating diagenesis (Figure 7). In the original samples, the Archaea Nitrososphaeria class dominated the shallower depths (2–5 cm and 7–10 cm) of the core, with a relative abundance of ~50%–70%. Its relative abundance decreased with depth and was barely found in the deeper sample (23–26 cm). After incubation, its relative abundance dropped to < 25%, highlighting the difficulty to cultivate these subsurface microorganisms. Nanoarchaeia was found in the deepest t_0_ sample and was nearly absent in the shallow part of the core as well as in the incubation samples. Members of the Bacteroidia class, which were barely observed in the core samples, dominated the incubation samples. One Bacteroidia ASV in particular represented 60.7%, 85.2%, and 37.4% of the sequences in *t*_2_‐5–7 cm, *t*_2_‐21–23 cm, and *t*_2_‐26–28 cm, respectively (Table S7). The Bacilli class relative abundance also increased from < 1% in the core samples to 2%–7% in the shallow sample incubations (Figure 7). The dominance of a few ASVs in the enrichments lowered the richness (*S*), Pielou's evenness (*J*), and the Shannon alpha diversity (Table S9). The Shannon diversity ranged from 4.01 to 6.02 in the core samples and from 1.03 to 4.19 in the incubated samples. Pielou's evenness ranged from 0.78 to 0.89 in the core samples and from 0.18 to 0.74 in the incubated samples.

### 3.4 Metal Concentrations and Microbial Community Composition Correlations

**FIGURE 8:**
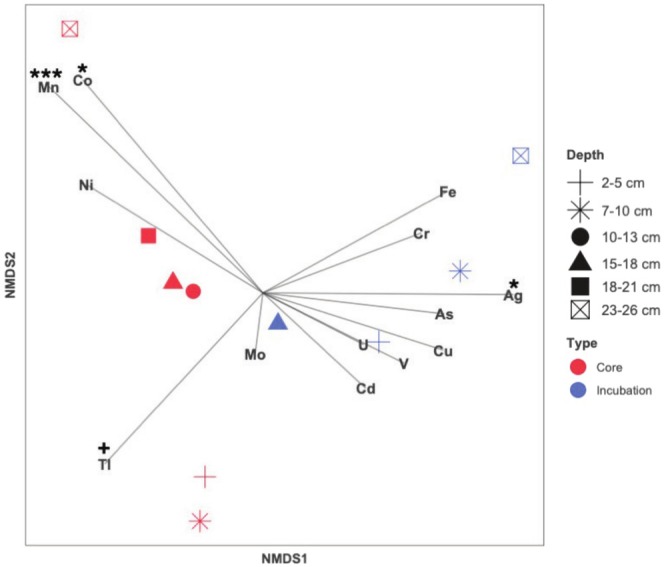
Non‐metric multidimensional scaling (NMDS) of *t*_0_ (red) and *t*_2_ (blue) samples from PS119_14‐1. Each measured metal is plotted as a vector, where the length and direction indicate the contribution of the variable to the principal component. Significant variables, as *p*‐values, are labeled as: *** = 0 < value ≤ 0.001, ** < 0.001, * < 0.01, and +< 0.05.

A non‐metric dimensional scaling (NMDS) analysis revealed that, in general, the samples cluster by treatment, initial core (*t*_0_) or incubated samples (*t*_2_) (Figure 8). The core samples also cluster by depth. Correlations between the metal concentrations and the microbial composition were identified to determine which factor was potentially driving the microbial assemblages. The metals that are correlated with the microbial composition of the samples are Mn (*r* ^2^ = 0.8626 and *p* = 0.001) Co (*r* ^2^ = 0.7513 and *p* = 0.011), Ag (*r* ^2^ = 0.6473 and *p* = 0.035), and, to a lesser significant correlation, Tl (*r* ^2^ = 0.5267 and *p* = 0.074) (Figure 8, Table S8).

**FIGURE 9:**
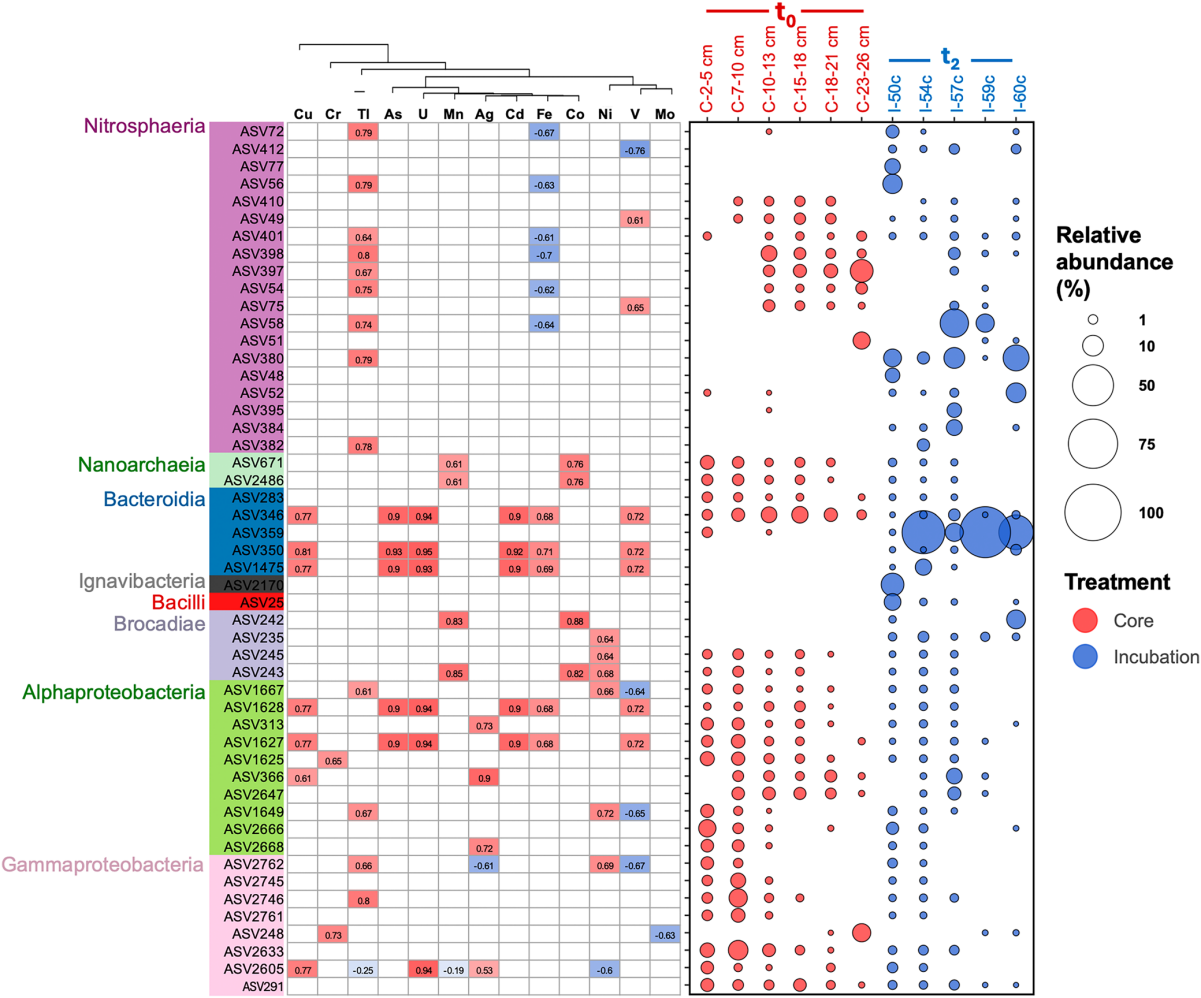
Correlation between each of the 50 most abundant ASV with the trace metal concentrations (left panel) and relative abundance in the core and incubation samples of each ASV (right panel) from PS119_14‐1 *t*_0_ (red circles; Core) and *t*_2_ (blue circles; Incubation) samples. Metals were clustered based on their concentrations. Only correlations with a *p*‐value < 0.05 are displayed.

The possible correlations between metal concentration and ASV relative abundance were evaluated (Figure 9). Some of the correlation patterns were similar among metals. Most correlation patterns were identified by microbial taxa. For example, most Nitrosphaeria ASVs were positively correlated with Tl and negatively correlated with Fe. Some of the most abundant Nitrosphaeria ASVs (e.g., ASV380) were only positively correlated with Tl. Most Brocadiae ASVs, regardless of their relative abundance in the core (*t*_0_) or incubations (*t*_8_), were positively correlated with Cu, As, U, Cd, Fe, and V.

## 4 Discussion

The observed, subtle TOC variations with depth at both sites in the initial samples likely indicate changes in productivity, potentially caused by nutrient delivery from volcanic ash sourced from the South Sandwich Island Volcanic Arc (Duggen et al. 2010; Bohrmann 2019). The low organic carbon contents (< 0.8 wt.%), coupled with high sedimentation rates of siliceous ooze, result in extended redox zonations at the investigated sites and provide an opportunity to investigate the fate of trace metals cycled during very early diagenesis in a natural system. The observed low (< 3 mM) and generally homogenous alkalinity values similar to seawater (~2.5 mM) (Tables S5 and S6) also indicate low metabolic rates in the sediments at the time of collection. This inference is supported by the low rates of (net) SO_4_^2−^ reduction as indicated by the lack of downcore decreases in SO_4_^2−^ concentrations. Interestingly, during the incubation experiment, SO_4_^2−^ values show a slight increase, particularly at the inflection point in the TOC profile for PS119_14‐1 (Figure 4A). Nearby hydrothermal vents can act as sources of sulfide nanoparticles (e.g., pyrite) to the investigated sites, surviving oxidation in the water column (Yücel et al. 2011). During diagenesis, these sulfides may be oxidized by microbial activity or abiotically, leading to the increases we observe in SO_4_^2−^ concentrations to values higher than the overlying seawater (bottom water values here are ~29 mM; Tables S5 and S6).

However, as the incubations were conducted in sealed bags under an anoxic atmosphere, the oxidation of sulfides is likely minimal. Since these sediments are closed systems, sulfide formation would likely rapidly draw down porewater SO_4_^2−^ and trace metals without the potential for replenishment by diffusion or advective fluid flow. The relatively stable SO_4_^2−^ values downcore and throughout the incubations thus support minimal oxidation effects. In future studies, analysis of porewater nutrients (e.g., phosphate), SO_4_^2−^, oxygen, and solid phase mineral formation, isotopic composition, and speciation is necessary to more fully understand the diagenetic cycles occurring in incubated marine sediments. Overall, our incubation experiments show a spatial and temporal variability of dissolved trace metal abundance continuously stimulating and challenging the microbial community to adapt and respond to environmental changes.

### 4.1 Dissolved Metal Behavior

The large input of ash and/or hydrothermally‐sourced Fe and Mn (oxyhydr)oxides from the ridge and island arc (Sander and Koschinsky 2016; Pereira et al. 2022), which are not observed in typical pelagic sediments, leads to a high delivery of trace metals to the seafloor by particle scavenging. Iron in the porewater is near or below detection limit at both sites at initial sampling and during the incubations (Tables S1 and S2) suggesting that the observed processes are limited to Mn reduction coupled to organic matter mineralization. The aforementioned indications of minimal SO_4_^2−^ reduction suggest that the low dissolved Fe contents are not attributable to uptake into sulfide minerals (Figures 5 and 6).

The lack of evidence for Fe reduction indicates an extended Mn reduction zone with depth, and the continued absence of significant porewater Fe concentrations in the incubations could suggest protracted Mn reduction through time. The highest Mn concentrations are observed in *t*_0_ samples and the uppermost t_1_ sample in PS119_15‐2, but otherwise, Mn (oxyhydr)oxide cycling is not directly indicated. Instead, correlations with various trace metals (Co, Ni, Cu, Mo, and Tl), as discussed below, which adsorb to the surface of oxide minerals may provide indirect evidence of ongoing Mn (oxyhydr)oxide reduction. This necessitates further study, but perhaps a process is occurring that resembles sulfur recycling during the cryptic sulfur (Canfield et al. 2010) and/or iron cycles (Kappler and Bryce 2017), where dissolved Mn is re‐adsorbing onto residual oxides, removing it from the porewater but leaving previously adsorbed metals in the dissolved phase. While there are statistically significant correlated changes in metal concentrations to changes in the microbial community in these samples, future studies could include sterilized sediments to monitor any metal cycling and geochemical processes that could be occurring in addition to the microbially driven biogeochemical cycles discussed here.

At first glance, porewater concentration profiles of Mn and trace metals seem more correlated in samples from PS119_15‐2 compared to PS119_14‐1. This is particularly apparent in the uppermost sample of incubation *t*_1_ (PS119_15‐2), where there is a clear and concomitant release of Co, Ni, Cu, Mo, Ag, Cd, and Tl with Mn. The correlation coefficients in this case are very high, but notably they are driven by only that one surface sample (Table S10). Samples from PS119_15‐2 at *t*_0_ also show a high level of correlation between Mn and Ni (*R* ^2^ = 0.9021), Mn and Co (*R* ^2^ = 0.9683), Mn and Cd (*R* ^2^ = 0.8105). The observed correlations significantly decrease at *t*_2_, with Mn and Ni (*R* ^2^ = 0.3116) and Mn and Cd (*R* ^2^ = 0.1615). At site PS119_14‐1, the correlations are less apparent (Table S10). At *t*_0_, only Mn and Co (*R* ^2^ = 0.7555) and Mn and Ni (*R* ^2^ = 0.5518) have *R* ^2^ values above 0.5, which decrease significantly by *t*_1_. The correlations at *t*_2_, although higher, are only driven by one sample. Interestingly, at *t*_1_, Ag (*R* ^2^ = 0.9219) and Cr (*R* ^2^ = 0.773) show significant correlation with Mn.

The discrepancy between the two sites could be caused by differences in porosity, permeability, residual minerals, and/or reaction timescales. In PS119_14‐1 sediments, Mn may be reprecipitating as oxides before trace metals (co‐)precipitate or adsorb. Trace metals can also compete for binding sites on authigenic minerals or residual Fe and Mn (oxyhydr)oxides (Wehrli and Stumm 1989; Manceau and Charlet 1992; Peacock and Sherman 2007; Wang et al. 2013). Differences in porewater behavior and diagenetic processes through time and between cores may also stem from depositional conditions. Core PS119_14‐1 is located on a topographic high while PS119_15‐2 was collected in a local topographic low; sedimentary hydrothermal input, grain size, and distribution could be slightly different in the two sites, and thus affect diagenetic rates by marginally altering porosity or permeability. Additionally, other early diagenetic processes such as silicate alteration or organic matter mineralization could explain the differences between Mn and other dissolved metals among these two sites.

The different groupings and patterns of metal release observed during our simulated diagenesis likely indicate a complex interplay of many sedimentary and biogeochemical variables. In t_0_ samples, Mn, Co, and to an extent Ni increase downcore, indicating Mn reduction occurring in the sediments releasing Co and Ni preferentially to other trace metals (Figures 5 and 6). For example, the grouping of Mn, Co, and Ni at t_0_ may indicate surficial adsorption of Co and Ni to the Mn particles, whereas the similar release in the uppermost sample of Mn, Co, Ni, Cu, Mo, Cd, and Tl in PS119_15‐2 at *t*_1_ may indicate either location of these metals in other phases (e.g., organic matter) or stronger adsorption on, or inclusion within, the oxide crystal lattice of these trace metals (Figures 5 and 6). Inclusion within the crystal lattice may require longer to release them from the Mn (and Fe) (oxyhydr)oxides than adsorption onto the surfaces which are often more susceptible to environmental changes and are the direct redox interface (e.g., Flynn and Catalano 2019).

As a specific example, in contrast to the previously described correlation between the alteration of Mn oxide minerals and V (Cui et al. 2023; Garcia‐Orozco et al. 2023), we do not observe a direct relationship in the porewater profiles of Mn and V as could be expected (Figures 5 and 6). This could be related to the increased availability of trace metals in our sediments and the competition of these metals during authigenic mineral formation and their differences in adsorption affinity and capacity relative to the oxide mineral structure (e.g., Wehrli and Stumm 1989; Manceau and Charlet 1992; Peacock and Sherman 2007; Wang et al. 2013). The structure of oxide minerals can have a strong impact on the trace metal release. V and Cu readily adsorb onto both Fe and Mn (oxyhydr)oxides, but Mo and Ni are associated only with Mn(oxyhydr)oxides and show little affinity to Fe(oxyhydr)oxides (Tribovillard et al. 2006 *and references therein*), the observed higher correlation coefficients of Mo and Ni with Mn, and the low correlation with V is consistent with our observations. Frierdich and Catalano (2012) demonstrated that specific incorporated metals such as Ni could be released from crystalline Fe‐oxide minerals in the presence of dissolved Fe without active iron reduction. The release of Ni is accomplished by electron transfer and atom exchange between aqueous Fe(II) and Fe(III) oxides, where Fe(II) undergoes oxidative adsorption and Fe(III) undergoes reductive dissolution, cycling Ni at the same time, though Fe(II) concentrations and pH affect the reaction rate and metal concentrations (Frierdich and Catalano 2012). Therefore, some of the Ni measured in the pore waters may be associated with these processes, complicating the correlation with Mn.

It is noteworthy that in systems such as the Scotia Sea with elevated Fe and Mn input, and despite indicated low metabolic rates, trace metal cycling could be even more pronounced compared to sediments with high organic matter input because the metals are not immediately consumed in authigenic sulfide minerals. Instead, the protracted redox zones which our incubations simulate could allow continued recycling of Fe, Mn, and trace metals as well as diffusion into the overlying water column.

### 4.2 Microbe–Dissolved Metal Interactions and Effects on Environmental Microbiology

To evaluate the effect of the released metals on the sediment biosphere, we statistically correlate changes in abundances of members of the microbial community, determined by 16S rRNA analyses, to changes in trace metal concentrations in incubated sediments. Using 16S rRNA analysis of the original and 8‐month incubated samples from core PS119_14‐1, we observe a decrease in diversity in the sedimentary microbial community through time, which is supported by the Simpson alpha diversity measure (Table S9). There is a shift in the microbial community composition from significant amounts of Nitrosphaeria at *t*_0_ to dominance of Bacteroidia at *t*_2_ (Figure 7). Shifts between these taxa in high latitude water columns have been previously correlated to changes in carbon sources, for example (Thiele et al. 2023), but we do not observe similar variations in organic matter content (Figure 4). While the shift in the microbial community could simply be from the system becoming more reducing as a result of the closed and anoxic incubation setup, the microbial assemblages only correlate with some trace metals. As Mn (oxyhydr)oxides are undergoing reduction, a host of trace metals are released into the porewater because of this redox shift (Figures 5 and 6), though only Mn, Co, Ag, and Tl are statistically significant for the microbial community (Figure 8). On a finer scale, Bacteroidia, which came to dominate the microbial community composition during the incubations, showed the strongest correlation with only Cu, As, U, Cd, Fe, and V (Figure 9). It is likely a combination of changes in redox conditions and metal cycling that drives the microbial assemblages.

The three trace metals with statistically significant correlations between ASVs and trace metal concentrations are Co, Ag, and Tl, all of which have been investigated as potentially toxic metals for microorganisms. Cobalt typically enters cells via non‐specific ion pumps, such as Mg^2+^ pumps (Paulo et al. 2017). Once inside the cell, Co can deactivate Fe‐S proteins, inhibit sulfur metabolism, and the creation of Fe‐S clusters, and cause reactive oxygen species to form (Eitinger 2013; Paulo et al. 2017). Similarly, Ag can lead to the creation of reactive oxygen species such as hydroxyl radicals but can also damage nucleic acids, bind with and inhibit protein activities, and affect cellular transport and respiration (Zhao et al. 2021). As Ag^+^, it has a strong affinity for negatively charged bacterial membranes, even leading to direct physical effects on cellular membranes, inhibiting growth (Zhang et al. 2018; Gordienko et al. 2019; Zhao et al. 2021). Concentrations of free Ag^+^ in the water column as low as ~1 μg/L have been shown to be toxic to a variety of organisms (Call et al. 2006). Thallium has been shown to be highly toxic in increased concentrations for microbial communities and other higher life forms (e.g., Norris et al. 1976; Liu et al. 2023). The effect of elevated dissolved Tl concentrations on benthic organisms is still largely unknown, though recent studies by Markich et al. (2023) investigating tropical and temperate marine organisms, including microorganisms, see an effect of Tl toxicity beginning at concentrations as low as 3 μg/L (~15 nM). In our study, Tl concentrations reached up to 14 nM, which is over an order of magnitude higher than bottom water concentrations (331 pM) and within the toxic range for benthic lifeforms (Markich et al. 2023).

Similarly to Mo, it has been shown that high dissolved concentrations of Cd (at μM level) can inhibit iron reduction and impact Fe(II) formation rates (Burkhardt et al. 2011). It has been shown in laboratory experiments that the addition of high (μM) concentrations of other metals, such as Cu, Ni, or Zn, inhibited or strongly reduced the formation of ferrous iron (Burkhardt et al. 2011). Although the Cd concentrations in our samples do not reach such high values (Figures 4 and 5), it cannot be excluded that the combined concentration of heavy metals released during the incubation experiment might impact specific microbial activities and rates and, with that, the sequence and extent of early diagenetic processes within the sediments. Even these lower Cd concentrations impact the microbial community composition in our sample. This is in good agreement with previous results from Antarctic, anthropogenically contaminated sites where such an impact by Cd was also implicated (Powell et al. 2003). Furthermore, trace metals in our samples are not being re‐precipitated in authigenic sulfides as evidenced by continued high concentrations of porewater SO_4_^2−^ throughout the incubations. Potential buildup in non‐euxinic porewaters, and thus, non‐sulfidic sediments may result in greater toxicity of metals such as Cd and Ni compared to their acute toxicity in sulfidic sediments previously investigated (Di Toro et al. 1992).

Our results may also provide insights into the origin and potential evolution of metabolic pathways in Earth's history. For example, methanogenesis, a crucial and ancient carbon cycling metabolism, requires Ni, while nitrogen fixation requires both Mo and Cu. We show that in organic‐poor Fe and Mn‐rich settings, these critical elements are significantly diagenetically cycled.

### 4.3 Comparison to Polluted Sites and Potential Fluxes of Trace Metals

Overall, our data show that in addition to the potential role of previously described carbon source changes in the Scotia Sea (e.g., Thiele et al. 2023), strong variations in metal concentration in the porewater can cause a significant shift in the microbial community. Interestingly, our analysis revealed that the metals that are highly statistically correlated with the ASVs, namely Co, Ag, and Tl, are also metals that have a high affinity towards Fe and Mn oxide mineral surfaces (Chao and Anderson 1974; Murray 1975; Owens et al. 2017). The statistical correlation observed between dissolved trace metal availability and the microbial community might be related to a competition between microbes and metals during mineral dissolution or authigenic mineral formation for the surface space as specifically benthic microbes have a high affinity towards particle surfaces (Cooksey and Wigglesworth‐Cooksey 1995; Ehrlich 1997). Indeed, Bacteroidia has adaptations for growth on particle surfaces, perhaps explaining its dominance in the incubated samples (Fernández‐Gómez et al. 2013). However, because of limitations in the incubation experimental setup, other processes that can affect the microbial community were not evaluated and should be considered in future experiments. For example, the sudden cutoff from oxygen and other nutrients that would be diffusing into the sediments from the overlying water column may have also affected the community. The starting microbial community might also not be in steady state due to effects from variations in hydrothermal vent activity, seasonal changes, or natural, non‐simulated diagenesis. Lower pressures relative to seafloor conditions may also affect the microbial community, though temperature is often the driving factor in incubation experiments of marine sediments (Perez Calderon et al. 2019). Future experiments could investigate these effects by sampling for geochemistry and microbiology at more frequent intervals, incorporating broader geochemical analyses including isotopes, tracers, and speciation, and storing samples in a variety of containers to eliminate the possibility of plastic degradation affecting the microbial community or adsorbing metals.

While previous studies have shown that certain metals can have strong impacts, both positive and negative, on the microbial community, most were carried out for non‐marine systems and/or as laboratory experiments focusing on single microbe‐element interactions (Sullivan and Yelton 1988; Gough and Stahl 2011; Kwon et al. 2015; Li et al. 2020). Even in marine systems, most studies have focused on polluted sites such as Southern Norway (Gillan et al. 2005), Pula Bay, Croatia (Di Cesare et al. 2020), and the East China Sea (Chen et al. 2022) and on solid phase trace metal content instead of porewater trace metal concentrations (Gillan et al. 2005; Di Cesare et al. 2020; Chen et al. 2022). Anthropogenic input of nutrients and metals in these studies is likely different from natural systems, and their diagenetic processes are not as limited by organic matter or nutrient supply as those in our study. In these organic matter‐rich settings, the sediments quickly progress towards SO_4_^2−^ reduction and eventual precipitation of authigenic sulfides, which often act as a sink for trace metals in marine sediments (Tribovillard et al. 2006, *and references therein*).

Even given these different depositional conditions and inputs, our samples generally have (naturally) elevated Cu content (~80 ppm) (Figure 3) in the solid phase relative to some polluted sites, which have < 50 ppm Cu (Powell et al. 2003; Gillan et al. 2005; Chen et al. 2022). Solid phase content of other metals (e.g., Ni, Cr, Cd, As) (Figure 3) is comparable to other polluted sites. The observed elevated Cu content in the sediments is a good indicator of hydrothermal input in this location, and its comparably high levels relative to documented polluted sites warrant further investigation into the role of hydrothermally sourced metals in controlling proximal benthic and sedimentary ecosystems. Mayor et al. (2013) discuss the multi‐level impacts and controls on Cu toxicity in sediments, emphasizing the role that macrofauna have on microbial communities and processes, primarily through the process of bioturbation. Elevated sedimentary Cu content has adverse effects on macro‐ and microfauna, and porewater cycling of Cu, which we observe, sourced from high‐Cu content sediments may have additional effects on microbial community composition. However, Cu, alongside Mo and Fe, is also a critical enzymatic cofactor in denitrification, a pervasive anaerobic metabolic pathway, highlighting that trace metals may inhibit some microbial communities while stimulating others (Wasser et al. 2002).

In depositional settings with high metal content but low TOC and low terrigenous input, large diagenetic releases of elements can impact the overlying benthic fauna and the water column (e.g., Johnson et al. 1999). In our incubations, Cu released into the porewater in the uppermost sediment layer exceeds 100 μM at both investigated sites (Figures 5 and 6) exceeding the binding capacity of Cu onto solids (Shaw et al. 1990). The accumulating dissolved Cu in the porewater can diffuse to less concentrated areas either downward, where Cu precipitates or adsorbs onto solids, or upward back into the water column (e.g., Koschinsky et al. 2001). Therefore, a benthic release of Cu into seawater may not be buffered by organic ligand complexation within the sediments, as we observe an accumulation in the porewaters. Potentially, this could allow sediment‐sourced Cu to diffuse into benthic marine ecosystems where organic ligand complexation may subsequently occur, which are often Cu limited (Ruacho et al. 2022). Similar to Cu, Tl, or other metals may be released during diagenesis, accumulate above binding capacities, and diffuse upwards into the water column, where they can impact benthic and planktonic communities (e.g., Riedel et al. 1997). In some settings, trace metals may be a limiting micronutrient (e.g., Morel and Price 2003); thus, a benthic flux upwards into the water column does not necessarily have negative implications for the marine environment.

## 5 Conclusions

Our experimental results provide snapshots into continued microbially induced marine trace metal cycling under changing redox conditions and electron donor availability in sediments with low organic matter and high Fe and Mn (oxyhydr)oxide content. In this location, hydrothermal and/or volcanic arc inputs source these oxides, but this general sedimentary environment may be extrapolated to many environments on Earth today, in its past, or on other worlds. The results show the remobilization of large amounts of trace metals within the sediments under changing redox conditions in these organic matter‐poor, metal‐rich systems under oxic overlying waters. Importantly, in previously studied heavy metal‐polluted ocean margin settings with high organic carbon (up to 11 wt.%, Pula Bay, Croatia) and nutrient input, changes in the microbial community were observed with the overall population driven by the nutrient availability (e.g., Giller et al. 1998; Di Cesare et al. 2020). In contrast, our analyzed sediments in the Scotia Sea have < 0.8 wt.% total organic carbon (TOC) with comparable or even higher metal content than previously studied polluted sediments. We show that at low nutrient availability and low TOC, the elevated release of heavy metals strongly impacts the microbial community, perhaps because in these settings metals are not quickly sequestered in authigenic sulfides. Importantly, our data also illustrate how continued biogeochemical processes can potentially alter signals from the time of sample collection to post‐cruise analysis.

By simulating diagenesis in a closed system, we have created an environment akin to the deep biosphere as well as geological settings that undergo changes in sediment input and/or redox conditions (e.g., the onset of Mn reduction in our samples) on short timescales, for example, fast burial (mass transport deposits; e.g., Hensen et al. 2003) or changes in hydrothermal vent activity. Our paired geochemical and microbiological results show that the release of elevated trace metal concentrations directly impacts the diversity and composition of the sedimentary microbial community and, with that, is an important physical and geochemical control on microbial metabolisms, activity, and processes in marine sediments. The change in community composition during the incubations is likely caused by the combined release of multiple metals (e.g., Mo, Cd Co, Cu, Ag, and Tl), though further integrated study of the involved microbial metabolic pathways, metagenomics, microbial detoxification mechanisms, trace metal cycles, and authigenic mineral formation pathways is necessary to fully understand these interactions. By understanding these processes and mechanisms, we will gain a better insight into marine sedimentary element cycling, mass balances, and microbial habitability and activity, especially in the deep biosphere.

Furthermore, by understanding the interaction of trace metals and microbes today, we can glimpse into how Earth's microbiome may have changed through time as metal content and availability have changed (Robbins et al. 2016; Moore et al. 2017). On geologic timescales, the order of release during diagenesis likely has a negligible impact on parameters such as paleoredox proxies, but we show that even small fluctuations in trace metal availability can affect microbial ecology in the sediments on shorter timescales. High metal content may create a feedback loop during diagenesis with metal release affecting metabolic rates and processes, further controlling diagenetic cycling and mineral dissolution rates (Welch and Vandevivere 1994). In some cases, these feedbacks in oxide‐rich, organic matter‐poor settings could result in benthic fluxes of metals across the sediment–water interface and into the water column. This has important implications for benthic and water column ecosystems as these metals may stimulate growth in metal‐limited systems or inhibit ecosystems when trace metals exceed toxicity limits. Indeed, a similar mechanism for early diagenetic nitrogen cycling has been described where buried microbial mats diagenetically release ammonium that can be oxidized at the sediment–water interface and incorporated into new biomass (Stüeken and Prave 2022). When viewed through time, the role of Fe and Mn (oxyhydr)oxide cycling and consequent controls on metal availability may have affected key metabolisms (Lyons et al. 2024). Depositional environments ranging from hydrothermally influenced, such as here, to ancient iron formations or Martian Fe oxide‐rich sediments may have similar trace metal cycles and thus similar biogeochemical feedbacks. Iron and Mn (oxyhydr)oxides phases could be a significant, beneficial source of metals to microbial systems in the water column or deep biosphere of Earth and beyond, particularly in SO_4_^2−^ limited environments where trace metals are not consumed during authigenic sulfide formation.

The observed shifts in microbial diversity reveal an important physical control on microbial ecology and diagenetic cycling of biologically relevant elements. The feedback between diagenetic and geochemical changes and the corresponding shift in the microbial community may give insight into the long‐term burial of Fe and Mn oxide‐rich sediments (e.g., banded iron formations, early oxygen oases, Martian sediments) and their role as sources of trace metals to deep biosphere life (Bell et al. 1990). While terrestrial hydrothermal settings differ from these deep time systems on Mars, the cycling of metals is likely similar. Further, in the absence of large‐scale iron formation deposits forming today on Earth, modern hydrothermal Fe and Mn deposits are some of the closest analogs for those ancient deposits. In particular, the large amounts of diatomaceous ooze could be an analog for the silica implicated in Archean iron formations. Similarly, by doing these incubations in a closed system, we simulate a restricted setting where trace metal availability may be more controlled by diagenetic reactions than by resupply from an overlying water column, representing a bottom‐up supply of nutrients and/or toxic metals (Sweere et al. 2016). In this study, we observed a complex cycle of metals, likely related to Mn (and Fe) (oxyhydr)oxide reduction. While we discuss the correlation between these metals and the in situ microbial community, additional work is needed in settings with different organic matter types and content, sediment input (e.g., carbonate sediments, detrital siliciclastics), and oceanographic environments (e.g., continental shelves, restricted basins).

Reconstructions of ancient environments often use redox‐sensitive trace metals as proxies for environmental conditions, redox, and productivity (Tribovillard et al. 2006 *and references therein*). However, the processes and feedbacks on metal deposition and sequestration in marine sediments are still not fully constrained. By studying the interaction between metals and the microbial community, we shed light on the role of biology in these geochemical cycles and how different members of the community respond. This can be extrapolated to evolving microbial communities through time, in particular ones that use the investigated trace metals in their metabolisms. Further study is necessary into the combined effects of multiple trace metals and changing metal availability during diagenesis, as variations with depth in these biogeochemical cycles could mimic changing metal availability through Earth's history. In particular, the cycling of Mo, Ni, and Cu is critical for understanding biogeochemical controls and factors in key metabolisms throughout Earth history such as methanogenesis (Ni) and nitrogen fixation (Mo, Cu). This and future incubation studies may give insight into the co‐evolution of microbial communities and their biogeochemical environments through time.

## Conflicts of Interest

The authors declare no conflicts of interest.

## Supporting information


Data S1.

## Data Availability

Sample processing, sequencing, and core amplicon data analysis were performed by the Earth Microbiome Project (www.earthmicrobiome.org), and all amplicon sequence data and metadata have been made public through the EMP data portal (qiita.microbio.me/emp). Geochemical data is supplied in the Supporting Information of this manuscript and will be uploaded to the data repository PANGAEA.
