# Supplementary material for: Impacts on Sedimentary Microbial Communities Related to Temporal Changes in Trace Metal Concentrations
Source: Geobiology. 2025 Jul 8;23(4):e70027. doi: 10.1111/gbi.70027 (PMC12238750; doi:10.1111/gbi.70027)
Supplement: Supplementary file 1 — Data S1. [file GBI-23-e70027-s001.docx]

**Supplementary Materials**

***Supplementary Table 1.*** Core PS119_14-1 porewater trace metal data of vanadium (V), chromium (Cr), manganese (Mn), iron (Fe), cobalt (Co), nickel (Ni), copper (Cu), arsenic (As), molybdenum (Mo), silver (Ag), cadmium (Cd), thallium (Tl), and uranium (U) for initial core samples (t_0_), 4 month incubations (t_1_), and 8 month incubations (t_2_). All data was produced on an inductively coupled plasma-mass spectrometer (ICP-MS) at Oklahoma State University except for t_0_ manganese (Mn) and iron (Fe) data generated at Oregon State University (OSU) via ICP-optical emission spectroscopy and photometrically on the ship during Expedition PS119, respectively. NIST Standard Reference Material 1643f was included for data accuracy, and the measured and reference values are provided.

| **14-1** | Depth | V | Cr | Mn OSU | Mn | Ship Fe | Fe | Co | Ni | Cu | As | Mo | Ag | Cd | Tl | U |
| --- | --- | --- | --- | --- | --- | --- | --- | --- | --- | --- | --- | --- | --- | --- | --- | --- |
| Sample # | cm | nM | nM | µM | µM | µM | µM | nM | nM | nM | nM | nM | nM | nM | pM | nM |
| **t_0_** |  | | | | | | | | | | | | | | | |
| 49 | 0.00 | 38.19 | 11.51 | 0.00 |  | 0.2 | 0.59 | 1.83 | 15.69 | 10.28 | 25.32 | 120.25 | 0.08 | 0.56 | 86.91 | 12.78 |
| 50 | 0.50 | 97.19 | 18.63 | 0.00 |  | 0.1 | 0.60 | 2.11 | 26.61 | 102.73 | 36.67 | 149.61 | 1.30 | 2.47 | 32.73 | 11.35 |
| 51 | 1.50 | 79.26 | 55.09 | 0.00 |  | 0.0 | 0.87 | 2.52 | 29.74 | 105.57 | 43.23 | 146.19 | 1.43 | 2.11 | 71.73 | 11.76 |
| 52 | 2.50 | 133.93 | 17.89 | 0.00 |  | 0.1 | 0.61 | 2.55 | 26.26 | 113.31 | 40.54 | 104.58 | 1.69 | 2.20 | 20.55 | 11.82 |
| 53 | 4.00 | 143.60 | 24.27 | 0.00 |  | 0.3 | 0.62 | 2.69 | 26.87 | 128.95 | 35.77 | 124.06 | 2.19 | 3.62 | 104.69 | 12.20 |
| 54 | 6.00 | 115.64 | 9.48 | 0.00 |  | 0.0 | 0.54 | 2.52 | 44.57 | 127.77 | 38.47 | 106.43 | 4.15 | 3.67 | 89.85 | 12.10 |
| 55 | 8.00 | 84.82 | 12.03 | 1.41 |  | 0.1 | 0.44 | 2.89 | 86.35 | 125.07 | 32.94 | 87.15 | 0.81 | 3.04 | 365.69 | 11.61 |
| 56 | 11.00 | 90.06 | 8.12 | 9.80 |  | 0.0 | 0.60 | 2.53 | 142.19 | 107.74 | 35.23 | 115.86 | 0.78 | 2.30 | 121.80 | 13.49 |
| 57 | 14.00 | 64.83 | 9.15 | 11.35 |  | 0.0 | 0.48 | 3.18 | 117.54 | 78.33 | 29.24 | 92.77 | 0.83 | 1.49 | 159.33 | 11.93 |
| 58 | 18.00 | 79.81 | 21.86 | 17.32 |  | 0.0 | 0.75 | 3.59 | 109.10 | 79.29 | 31.33 | 103.78 | 0.44 | 2.16 | 234.45 | 11.57 |
| 59 | 22.00 | 92.28 | 6.47 | 22.30 |  | 0.0 | 0.98 | 3.61 | 106.44 | 94.21 | 41.09 | 97.49 | 0.69 | 1.97 | 99.83 | 11.65 |
| 60 | 27.00 | 84.74 | 8.94 | 27.01 |  | 0.0 | 0.63 | 6.41 | 100.74 | 76.83 | 34.05 | 94.37 | 0.40 | 1.44 | 101.49 | 11.21 |
| 61 | 31.00 | 95.89 | 6.66 | 28.63 |  | 0.1 | 0.67 | 7.72 | 111.16 | 77.64 | 33.72 | 95.93 | 0.35 | 1.53 | 66.63 | 11.39 |
| **t_1_** |  | | | | | | | | | | | | | | | |
| 50b | 0.50 | 196.43 | 10.85 |  | 0.07 |  | 0.20 | 3.30 | 34.08 | 424.63 | 107.04 | 221.07 | 9.01 | 11.39 | 2387.67 | 27.95 |
| 54b | 6.00 | 122.27 | 10.48 |  | 0.01 |  | 0.06 | 2.51 | 13.42 | 231.30 | 56.38 | 199.07 | 16.61 | 3.14 | 381.38 | 14.32 |
| 57b | 14.00 | 42.67 | 159.33 |  | 0.11 |  | 0.08 | 2.73 | 0.79 | 154.60 | 36.66 | 23.14 | 1.39 | 0.10 | 274.36 | 6.67 |
| 59b | 22.00 | 112.83 | 200.16 |  | 0.14 |  | 0.93 | 3.00 | 15.31 | 128.07 | 43.08 | 70.33 | 1.77 | 0.64 | 372.85 | 9.67 |
| 60b | 27.00 | 134.59 | 125.41 |  | 0.12 |  | 0.19 | 2.85 | 27.79 | 117.11 | 50.57 | 22.94 | 1.43 | 0.81 | 74.22 | 7.45 |
| t_2_ |  | | | | | | | | | | | | | | | |
| 50c | 0.50 | 171.54 | 24.23 |  | 1.26 |  | 1.05 | 3.23 | 65.68 | 647.52 | 104.73 | 173.08 | 9.83 | 12.73 | 0.63 | 43.83 |
| 54c | 6.00 | 119.41 | 12.80 |  | 0.01 |  | 0.79 | 2.58 | 47.60 | 449.49 | 64.67 | 157.24 | 12.11 | 5.75 | 0.23 | 21.05 |
| 57c | 14.00 | 84.02 | 277.87 |  | 0.16 |  | 0.81 | 2.41 | 29.33 | 345.31 | 42.74 | 0.00 | 1.67 | 0.89 | 0.00 | 9.15 |
| 59c | 22.00 | 111.30 | 278.93 |  | 0.01 |  | 0.83 | 2.63 | 49.37 | 133.15 | 45.08 | 24.38 | 5.17 | 0.74 | 0.21 | 7.93 |
| 60c | 27.00 | 124.24 | 227.27 |  | 0.04 |  | 0.77 | 2.17 | 31.29 | 123.29 | 43.38 | 8.46 | 1.89 | 0.56 | 0.00 | 7.35 |
| NIST 1643f (Measured) | - | 34.20 | 16.81 |  | 37.05 |  | 95.84 | 23.07 | 57.06 | 20.30 | 52.92 | 104.45 | - | 5.47 | - | - |
| NIST 1643f (Reference) | - | 35.71 | 18.32 |  | 36.77 |  | 92.51 | 25.05 | 59.2 | 21.44 | 56.85 | 114.2 | - | 5.83 | - | - |

***Supplementary Table 2.*** Core PS119_15-2 porewater trace metal data of vanadium (V), chromium (Cr), manganese (Mn), iron (Fe), cobalt (Co), nickel (Ni), copper (Cu), arsenic (As), molybdenum (Mo), silver (Ag), cadmium (Cd), thallium (Tl), and uranium (U) for initial core samples (t_0_), 4 month incubations (t_1_), and 8 month incubations (t_2_). All data was produced on an inductively coupled plasma-mass spectrometer (ICP-MS) at Oklahoma State University except for t_0_ manganese (Mn) and iron (Fe) data generated at Oregon State University (OSU) via ICP-optical emission spectroscopy and photometrically on the ship during Expedition PS119, respectively. NIST Standard Reference Material 1643f was included for data accuracy, and the measured and reference values are provided.

| **15-2** | Depth | V | Cr | Mn OSU | Mn | Ship Fe | Fe | Co | Ni | Cu | As | Mo | Ag | Cd | Tl | U |
| --- | --- | --- | --- | --- | --- | --- | --- | --- | --- | --- | --- | --- | --- | --- | --- | --- |
| Sample # | cm | nM | nM | µM | µM | µM | µM | nM | nM | nM | nM | nM | nM | nM | pM | nM |
| **t_0_** |  | | | | | | | | | | | | | | | |
| 78 | 0.00 | 36.92 | 14.84 | 0.00 |  | 0.0 | 0.61 | 1.98 | 8.15 | 6.95 | 23.75 | 119.15 | 0.10 | 0.81 | 331.70 | 12.20 |
| 79 | 0.50 | 133.64 | 24.93 | 0.00 |  | 0.0 | 0.57 | 2.62 | 18.72 | 118.66 | 42.61 | 99.14 | 0.67 | 1.45 | 70.16 | 11.03 |
| 80 | 1.50 | 177.92 | 10.29 | 1.41 |  | 0.0 | 0.53 | 2.38 | 39.32 | 92.65 | 39.56 | 109.97 | 4.15 | 1.75 | 60.14 | 10.84 |
| 81 | 2.50 | 118.75 | 24.70 | 0.00 |  | 0.0 | 0.58 | 2.98 | 12.75 | 102.30 | 44.38 | 105.84 | 6.56 | 1.81 | 35.45 | 12.30 |
| 82 | 4.00 | 98.21 | 11.88 | 0.00 |  | 0.0 | 0.67 | 2.32 | 14.70 | 123.24 | 31.14 | 99.97 | 5.63 | 2.04 | 33.92 | 12.36 |
| 83 | 6.00 | 65.08 | 11.18 | 0.00 |  | 0.0 | 0.45 | 1.94 | 7.15 | 74.86 | 37.13 | 102.69 | 6.29 | 2.20 | 271.39 | 12.16 |
| 84 | 8.00 | 71.69 | 21.34 | 0.00 |  | 0.0 | 0.56 | 2.31 | 10.14 | 84.08 | 33.67 | 76.78 | 1.48 | 2.20 | 0.00 | 11.67 |
| 85 | 11.00 | 61.58 | 9.54 | 0.00 |  | 0.0 | 0.42 | 3.45 | 3.81 | 81.26 | 42.11 | 65.41 | 0.38 | 2.34 | 266.47 | 12.07 |
| 86 | 14.00 |  |  | 2.24 |  | 0.0 |  |  |  |  |  |  |  |  |  |  |
| 87 | 18.00 | 134.16 | 6.82 | 10.48 |  | 0.0 | 0.60 | 18.62 | 90.45 | 126.46 | 36.42 | 72.76 | 1.02 | 3.23 | 79.19 | 13.56 |
| 88 | 22.00 | 99.49 | 7.14 | 24.70 |  | 0.0 | 0.38 | 31.21 | 102.61 | 85.21 | 46.18 | 68.63 | 0.78 | 3.78 | 543.20 | 12.15 |
| 89 | 27.00 | 80.35 | 12.58 | 33.35 |  | 0.0 | 0.57 | 63.72 | 130.64 | 92.44 | 46.62 | 65.58 | 0.67 | 5.44 | 93.51 | 13.57 |
| 90 | 32.00 | 63.55 | 6.60 | 50.00 |  | 0.0 | 0.61 | 92.42 | 143.51 | 91.81 | 56.44 | 79.90 | 0.42 | 4.40 | 165.59 | 13.81 |
| 91 | 37.00 | 47.04 | 13.43 | 39.85 |  | 0.0 | 0.44 | 88.44 | 122.41 | 81.21 | 50.70 | 85.64 | 0.37 | 5.30 | 271.23 | 14.16 |
| **t_1_** |  | | | | | | | | | | | | | | | |
| 79b | 0.50 | 111.70 | 7.99 |  | 984.05 |  | 0.51 | 213.78 | 2309.66 | 987.58 | 108.35 | 2175.67 | 3.05 | 27.16 | 14409.58 | 31.18 |
| 83b | 6.00 | 134.97 | 21.43 |  | 0.12 |  | 0.49 | 2.37 | 4.30 | 177.13 | 59.10 | 213.76 | 12.91 | 2.85 | 329.56 | 16.47 |
| 86b | 14.00 | 101.26 | 42.80 |  | 0.08 |  | 0.49 | 13.59 | 17.59 | 214.06 | 50.66 | 72.10 | 1.00 | 1.03 | 130.17 | 8.78 |
| 88b | 22.00 | 166.40 | 37.95 |  | 0.25 |  | 0.90 | 3.04 | 42.04 | 189.24 | 53.90 | 58.59 | 2.59 | 1.34 | 19.49 | 9.54 |
| 89b | 27.00 | 169.78 | 96.44 |  | 0.11 |  | 0.42 | 1.67 | 17.42 | 167.16 | 47.81 | 45.72 | 1.65 | 0.47 | 560.34 | 7.74 |
| **t_2_** |  | | | | | | | | | | | | | | | |
| 79c | 0.50 | 155.93 | 24.13 |  | 0.01 |  | 0.84 | 3.51 | 67.79 | 570.56 | 100.78 | 194.68 | 33.73 | 5.75 | 1.68 | 42.66 |
| 83c | 6.00 | 148.32 | 18.24 |  | 0.39 |  | 0.79 | 3.06 | 21.43 | 310.73 | 70.02 | 139.53 | 7.32 | 2.97 | 0.00 | 20.18 |
| 86c | 14.00 | 131.22 | 47.02 |  | 0.06 |  | 0.86 | 2.82 | 49.15 | 338.47 | 53.27 | 58.88 | 1.80 | 3.71 | 0.00 | 9.07 |
| 88c | 22.00 | 158.10 | 26.04 |  | 0.45 |  | 0.76 | 2.52 | 54.30 | 227.60 | 48.26 | 37.87 | 1.67 | 1.21 | 0.07 | 13.30 |
| 89c | 27.00 | 200.55 | 155.75 |  | 0.01 |  | 0.74 | 2.83 | 51.98 | 213.22 | 58.73 | 49.01 | 2.05 | 0.93 | 0.14 | 10.47 |
| NIST 1643f (Measured) | - | 34.20 | 16.81 |  | 37.05 |  | 95.84 | 23.07 | 57.06 | 20.30 | 52.92 | 104.45 | - | 5.47 | - | - |
| NIST 1643f (Reference) | - | 35.71 | 18.32 |  | 36.77 |  | 92.51 | 25.05 | 59.2 | 21.44 | 56.85 | 114.2 | - | 5.83 | - | - |

***Supplementary Table 3.*** Core PS119_14-1 solid phase trace metal data of vanadium (V), chromium (Cr), cobalt (Co), nickel (Ni), copper (Cu), arsenic (As), molybdenum (Mo), cadmium (Cd), thallium (Tl), and uranium (U) for the initial core samples (t_0_), 4 month incubations (t_1_), and 8 month incubations (t_2_). All data was produced on an inductively coupled plasma-mass spectrometer (ICP-MS) at the University of California, Riverside. NIST Standard Reference Material 2702 was included for data accuracy, and the measured and reference values are provided.

| **14-1** | Depth | V | Cr | Co | Ni | Cu | As | Mo | Cd | Tl | U |
| --- | --- | --- | --- | --- | --- | --- | --- | --- | --- | --- | --- |
| Sample # | cm | ppm | ppm | ppm | ppm | ppm | ppm | ppm | ppm | ppm | ppm |
| **t0** |  |  |  |  |  |  |  |  |  |  |  |
| 49 | 0.00 |  |  |  |  |  |  |  |  |  |  |
| 50 | 0.50 | 58.41 | 18.54 | 8.81 | 21.32 | 73.25 | 2.32 | 0.46 | 0.46 | BDL | 0.93 |
| 51 | 1.50 | 58.35 | 12.01 | 8.58 | 22.31 | 72.65 | 2.86 | 0.57 | 0.57 | BDL | 0.57 |
| 52 | 2.50 | 59.94 | 21.58 | 9.11 | 25.42 | 76.25 | 2.88 | 1.44 | 0.48 | BDL | 0.96 |
| 53 | 4.00 | 73.06 | 25.57 | 11.42 | 32.42 | 85.84 | 3.20 | 2.28 | 0.46 | BDL | 0.91 |
| 54 | 6.00 | 55.17 | 19.91 | 7.39 | 18.20 | 66.55 | 2.28 | 1.14 | 0.57 | BDL | 0.57 |
| 55 | 8.00 | 65.46 | 22.79 | 11.15 | 29.58 | 83.89 | 3.39 | 2.42 | 0.48 | BDL | 0.97 |
| 56 | 11.00 | 59.97 | 18.56 | 9.04 | 19.04 | 75.68 | 2.38 | 1.90 | 0.48 | BDL | 0.95 |
| 57 | 14.00 | 63.33 | 21.11 | 9.82 | 15.71 | 77.08 | 2.95 | BDL | 0.49 | BDL | 0.49 |
| 58 | 18.00 | 65.45 | 14.35 | 11.48 | 17.80 | 88.41 | 3.44 | 0.57 | 0.57 | BDL | 0.57 |
| 59 | 22.00 | 76.72 | 19.06 | 15.15 | 16.61 | 103.59 | 4.89 | 0.98 | 0.49 | BDL | 0.49 |
| 60 | 27.00 | 69.06 | 10.39 | 9.87 | 9.87 | 76.33 | 2.08 | BDL | 0.00 | BDL | 0.52 |
| 61 | 31.00 | 77.87 | 21.11 | 7.51 | 11.73 | 85.85 | 2.35 | 0.00 | 0.47 | BDL | 0.94 |
| **t1** |  |  |  |  |  |  |  |  |  |  |  |
| 50b | 0.50 | 52.12 | 19.20 | 7.77 | 16.46 | 65.84 | 2.74 | 0.46 | 0.46 | BDL | 0.91 |
| 54b | 6.00 | 61.79 | 18.08 | 9.54 | 21.10 | 76.86 | 2.51 | 0.50 | 0.50 | BDL | 1.00 |
| 57b | 14.00 | 64.60 | 17.03 | 10.02 | 22.03 | 80.63 | 3.00 | 1.00 | 0.50 | BDL | 0.50 |
| 59b | 22.00 | 64.62 | 9.23 | 14.31 | 14.77 | 93.24 | 4.15 | 1.38 | 0.46 | BDL | 0.46 |
| 60b | 27.00 | 66.80 | 15.38 | 11.34 | 8.10 | 84.61 | 2.83 | BDL | 0.40 | BDL | 0.40 |
| **t2** |  |  |  |  |  |  |  |  |  |  |  |
| 50c | 0.50 | 50.84 | 17.94 | 7.48 | 17.45 | 64.80 | 1.99 | 0.50 | 0.50 | BDL | 0.50 |
| 54c | 6.00 | 62.18 | 22.06 | 9.53 | 18.05 | 72.71 | 2.51 | 0.50 | 0.50 | BDL | 1.00 |
| 57c | 14.00 | 60.41 | 19.97 | 9.73 | 14.85 | 76.28 | 3.07 | BDL | 0.51 | BDL | 0.51 |
| 59c | 22.00 | 62.14 | 13.11 | 13.11 | 10.68 | 81.56 | 2.91 | 0.49 | 0.49 | BDL | 0.49 |
| 60c | 27.00 | 70.92 | 21.00 | 9.33 | 10.73 | 80.25 | 2.80 | 0.00 | 0.47 | BDL | 0.47 |
| SRM 2702 (Measured) | - | 374.6 | 354.25 | 26.90 | 77.14 | 123.84 | - | 9.64 | - | - | 10.12 |
| SRM 2702 (Reference) | - | 357.6 | 352 | 27.76 | 75.40 | 117.70 | - | 10.80 | - | - | 10.40 |

***Supplementary Table 4.*** Core PS119_15-2 solid phase trace metal data of vanadium (V), chromium (Cr), cobalt (Co), nickel (Ni), copper (Cu), arsenic (As), molybdenum (Mo), cadmium (Cd), thallium (Tl), and uranium (U) for the initial core samples (t_0_), 4 month incubations (t_1_), and 8 month incubations (t_2_). All data was produced on an inductively coupled plasma-mass spectrometer (ICP-MS) at the University of California, Riverside. NIST Standard Reference Material 2702 was included for data accuracy, and the measured and reference values are provided.

| **15-2** | Depth | V | Cr | Co | Ni | Cu | As | Mo | Cd | Tl | U |
| --- | --- | --- | --- | --- | --- | --- | --- | --- | --- | --- | --- |
| Sample # | cm | ppm | ppm | ppm | ppm | ppm | ppm | ppm | ppm | ppm | ppm |
| **t0** |  |  |  |  |  |  |  |  |  |  |  |
| 78 | 0.00 |  |  |  |  |  |  |  |  |  |  |
| 79 | 0.50 | 61.03 | 21.86 | 9.56 | 23.23 | 76.52 | 3.19 | 0.46 | 0.46 | BDL | 0.91 |
| 80 | 1.50 | 58.98 | 21.63 | 9.34 | 24.08 | 77.66 | 2.95 | 1.47 | 0.49 | BDL | 0.98 |
| 81 | 2.50 | 61.56 | 18.90 | 9.69 | 25.69 | 78.52 | 2.91 | 1.94 | 0.48 | BDL | 0.97 |
| 82 | 4.00 | 59.70 | 11.73 | 10.13 | 27.19 | 78.36 | 2.13 | 1.60 | 0.53 | BDL | 1.07 |
| 83 | 6.00 | 62.79 | 22.05 | 10.54 | 28.76 | 79.56 | 2.88 | 3.83 | 0.48 | BDL | 0.96 |
| 84 | 8.00 | 61.96 | 9.01 | 11.27 | 34.92 | 83.36 | 2.25 | 8.45 | 0.56 | BDL | 0.56 |
| 85 | 11.00 | 57.08 | 20.32 | 8.71 | 11.13 | 83.20 | 1.45 | 0.48 | 0.48 | BDL | 0.97 |
| 86 | 14.00 | 72.02 | 25.01 | 7.00 | 11.00 | 85.02 | 1.50 | BDL | 0.50 | BDL | 1.00 |
| 87 | 18.00 | 70.66 | 11.68 | 7.23 | 11.13 | 74.00 | 1.67 | BDL | 0.56 | BDL | 0.56 |
| 88 | 22.00 | 70.81 | 21.75 | 25.29 | 13.66 | 99.13 | 3.03 | 0.51 | 0.51 | BDL | 0.51 |
| 89 | 27.00 | 67.83 | 16.58 | 11.56 | 8.54 | 91.44 | 3.01 | BDL | 0.50 | BDL | 0.50 |
| 90 | 32.00 | 78.91 | 26.14 | 9.05 | 13.07 | 95.00 | 1.51 | BDL | 0.50 | BDL | 0.50 |
| 91 | 37.00 | 68.44 | 20.87 | 7.77 | 15.05 | 76.69 | 1.46 | 0.49 | 0.49 | BDL | 0.49 |
| **t1** |  |  |  |  |  |  |  |  |  |  |  |
| 79b | 0.50 | 64.13 | 22.28 | 10.33 | 26.09 | 83.70 | 3.26 | 1.09 | 0.54 | BDL | 1.09 |
| 83b | 6.00 | 57.57 | 22.19 | 10.79 | 27.59 | 77.96 | 2.40 | 1.80 | 0.60 | BDL | 0.60 |
| 86b | 14.00 | 63.23 | 11.04 | 6.52 | 11.04 | 79.79 | 1.51 | BDL | 0.50 | BDL | 1.00 |
| 88b | 22.00 | 60.71 | 19.66 | 9.25 | 9.25 | 81.53 | 2.31 | BDL | 0.58 | BDL | 0.58 |
| 89b | 27.00 | 65.70 | 10.32 | 15.49 | 7.04 | 91.52 | 3.75 | 0.47 | 0.47 | BDL | 0.47 |
| **t2** |  |  |  |  |  |  |  |  |  |  |  |
| 79c | 0.50 | 59.56 | 19.45 | 9.12 | 25.52 | 77.79 | 2.43 | 1.82 | 0.61 | BDL | 0.61 |
| 83c | 6.00 | 57.91 | 8.27 | 10.34 | 19.65 | 71.36 | 2.07 | 2.59 | 0.52 | BDL | 0.52 |
| 86c | 14.00 | 61.19 | 10.67 | 6.74 | 11.23 | 61.19 | 2.81 | BDL | 0.56 | BDL | 0.56 |
| 88c | 22.00 | 59.22 | 15.56 | 8.21 | 9.08 | 76.95 | 1.73 | BDL | 0.43 | BDL | 0.43 |
| 89c | 27.00 | 65.24 | 5.02 | 17.29 | 5.02 | 95.35 | 2.79 | BDL | 0.56 | BDL | BDL |
| SRM 2702 (Measured) | - | 374.6 | 354.25 | 26.90 | 77.14 | 123.84 | - | 9.64 | - | - | 10.12 |
| SRM 2702 (Reference) | - | 357.6 | 352 | 27.76 | 75.40 | 117.70 | - | 10.80 | - | - | 10.40 |

***Supplementary Table 5.*** Core PS119_14-1 total organic carbon (TOC) and porewater sulfate (SO_4_^2-^) for initial core samples (t_0_), 4 month incubations (t_1_), and 8 month incubations (t_2_).

| **14-1** | TOC | SO_4_^2-^ | Alk |
| --- | --- | --- | --- |
| Sample # | wt% | mM | mM |
| **t_0_** |  | |  |
| 49 |  | 29.10 | 2.61 |
| 50 | 0.71 | 29.37 | 2.62 |
| 51 | 0.54 | 29.60 | 2.55 |
| 52 | 0.59 | 29.92 | 2.56 |
| 53 | 0.54 | 29.08 | 2.68 |
| 54 | 0.61 | 29.31 | 2.61 |
| 55 | 0.47 | 29.03 | 2.69 |
| 56 | 0.51 | 29.02 | 2.70 |
| 57 | 0.42 | 30.56 | 2.68 |
| 58 | 0.28 | 28.67 | 2.71 |
| 59 | 0.23 | 29.00 | 2.72 |
| 60 | 0.27 | 29.13 | 2.70 |
| 61 | 0.32 | 28.33 | 2.69 |
| **t_1_** |  | |  |
| 50b | 0.62 | 29.02 |  |
| 54b | 0.48 | 29.48 |  |
| 57b | 0.40 | 31.07 |  |
| 59b | 0.17 | 30.71 |  |
| 60b | 0.24 | 29.78 |  |
| **t_2_** |  | |  |
| 50c | 0.60 | 31.12 |  |
| 54c | 0.43 | 29.66 |  |
| 57c | 0.34 | 29.28 |  |
| 59c | 0.14 | 33.81 |  |
| 60c | 0.23 | 29.03 |  |

**Supplementary Table 6:** Core PS119_15-2 total organic carbon (TOC) and porewater sulfate (SO_4_^2-^) for initial core samples (t_0_), 4-month incubations (t_1_), and 8-month incubations (t_2_).

| **15-2** | TOC | SO_4_^2-^ | Alk |
| --- | --- | --- | --- |
| Sample # | wt% | mM | mM |
| **t_0_** |  | |  |
| 78 |  | 29.97 | 2.56 |
| 79 | 0.60 | 29.44 | 2.55 |
| 80 | 0.54 | 29.84 | 2.58 |
| 81 | 0.48 | 30.02 | 2.51 |
| 82 | 0.40 | 29.79 | 2.50 |
| 83 | 0.43 | 29.37 | 2.53 |
| 84 | 0.42 | 29.68 | 2.55 |
| 85 |  | 30.05 | 2.53 |
| 86 | 0.39 | 31.53 | 2.61 |
| 87 | 0.31 | 29.66 | 2.74 |
| 88 | 0.25 | 29.95 | 2.78 |
| 89 | 0.21 | 30.40 | 2.79 |
| 90 | 0.26 | 29.60 | 2.79 |
| 91 | 0.30 | 30.00 | 2.74 |
| **t_1_** |  | |  |
| 79b | 0.54 | 29.36 |  |
| 83b |  | 29.39 |  |
| 86b | 0.36 | 30.91 |  |
| 88b | 0.29 | 29.78 |  |
| 89b | 0.15 | 30.16 |  |
| **t_2_** |  | |  |
| 79c | 0.46 | 29.40 |  |
| 83c | 0.42 | 29.30 |  |
| 86c | 0.31 | 29.24 |  |
| 88c | 0.26 | 29.00 |  |
| 89c | 0.09 | 29.15 |  |

***Supplementary*** ***Table 7.*** Number of sequences from the sequencing (input), after filtration of of the sequences with Ns (filtered), denoising of duplicates (denoisedF for the forward sequence and denoisedR for the reverse sequence), merging of the forward and reverse sequences (merged), removal of the chimeras (nonchim), and clustering into ASVs.

|  | # reads | # filtered reads | # denoised reads (forward) | # denoised reads (reverse) | # merged sequences | # sequences with no chimera |
| --- | --- | --- | --- | --- | --- | --- |
| t_0_-02-5 cm | 12747 | 12369 | 11227 | 11490 | 7011 | 5907 |
| t_0_-07-10 cm | 9039 | 8032 | 7463 | 7544 | 4829 | 4352 |
| t_0_-10-13 cm | 51182 | 46978 | 44408 | 44765 | 33082 | 30787 |
| t_0_-15-18 cm | 22219 | 19891 | 17927 | 18142 | 13233 | 12617 |
| t_0_-18-21 cm | 54625 | 53641 | 51468 | 51573 | 40879 | 39461 |
| t_0_-23-26 cm | 24696 | 24235 | 23585 | 23358 | 20770 | 20530 |
| t_2_-0-1 cm | 42876 | 41990 | 40597 | 40776 | 33861 | 29231 |
| t_2_-5-7 cm | 81323 | 76996 | 75401 | 75635 | 68439 | 62924 |
| t_2_-13-15 cm | 19745 | 18409 | 17568 | 17576 | 14558 | 13587 |
| t_2_-21-23 cm | 99028 | 96643 | 95974 | 95857 | 92601 | 92176 |
| t_2_-26-28 cm | 64799 | 62164 | 61109 | 61045 | 56619 | 45270 |
| N-Control | 10477 | 10307 | 10294 | 10287 | 10279 | 10263 |

***Supplementary Table 8.*** Non-metric multidimensional scaling (NMDS) vector coordinate and strength (r^2^ and probability). Significant variables are labeled as: 0 ‘***’ 0.001 ‘**’ 0.01 ‘*’, and 0.05 ‘+’.

|  | NMDS1 | NMDS2 | r^2^ | Pr(>r) | Significance |
| --- | --- | --- | --- | --- | --- |
| Mn | -0.73891 | 0.6738 | 0.8626 | 0.001 | *** |
| Co | -0.66663 | 0.74539 | 0.7513 | 0.011 | * |
| Ag | 0.99998 | -0.00637 | 0.6473 | 0.035 | * |
| Tl | -0.6986 | -0.71551 | 0.5267 | 0.074 | . |
| Fe | 0.88686 | 0.46205 | 0.4479 | 0.12 |  |
| Ni | -0.86443 | 0.50275 | 0.4299 | 0.148 |  |
| Cr | 0.94174 | 0.33635 | 0.2915 | 0.184 |  |
| Cu | 0.958 | -0.28677 | 0.3653 | 0.202 |  |
| As | 0.9941 | -0.10844 | 0.3435 | 0.236 |  |
| V | 0.90542 | -0.42451 | 0.2511 | 0.361 |  |
| Cd | 0.75282 | -0.65822 | 0.1935 | 0.425 |  |
| U | 0.90058 | -0.43468 | 0.1292 | 0.568 |  |
| Mo | -0.13289 | -0.99113 | 0.0345 | 0.879 |  |

***Supplementary Table 9.*** Diversity measures for each core (t_0_) and incubated (t_2_) samples.

|  | **Shannon diversity (H)** | **Inv. Simpson** | **Simpson** | **Richness (S)** | **Pielou's evenness (J)** |
| --- | --- | --- | --- | --- | --- |
| t_0_-02-5 cm | 4.33 | 51.81 | 0.98 | 130 | 0.89 |
| t_0_-07-10 cm | 4.01 | 35.35 | 0.97 | 96 | 0.88 |
| t_0_-10-13 cm | 5.54 | 86.97 | 0.99 | 762 | 0.83 |
| t_0_-15-18 cm | 5.29 | 90.26 | 0.99 | 440 | 0.87 |
| t_0_-18-21 cm | 6.02 | 143.85 | 0.99 | 1023 | 0.87 |
| t_0_-23-26 cm | 4.91 | 32.08 | 0.97 | 522 | 0.78 |
| t_2_-0-1 cm | 4.19 | 23.83 | 0.96 | 294 | 0.74 |
| t_2_-5-7 cm | 2.59 | 3.01 | 0.67 | 307 | 0.45 |
| t_2_-13-15 cm | 3.42 | 11.69 | 0.91 | 177 | 0.66 |
| t_2_-21-23 cm | 1.03 | 1.45 | 0.31 | 280 | 0.18 |
| t_2_-26-28 cm | 2.44 | 4.69 | 0.79 | 230 | 0.45 |

*Supplementary Table 10.* Correlation values between PS119_14-1 and PS119_15-2 porewater manganese (Mn) and metal concentrations of vanadium (V), chromium (Cr), cobalt (Co), nickel (Ni), copper (Cu), arsenic (As), molybdenum (Mo), cadmium (Cd), thallium (Tl), and uranium (U) for the initial core samples (t_0_), 4 month incubations (t_1_), and 8 month incubations (t_2_).

|  | V | Cr | Fe | Co | Ni | Cu | As | Mo | Ag | Cd | Tl | U |
| --- | --- | --- | --- | --- | --- | --- | --- | --- | --- | --- | --- | --- |
| **PS119_14-1** |  |  |  |  |  |  |  |  |  |  |  |  |
| Mn (t_0_) | 0.0324 | 0.1811 | 0.1212 | 0.7555 | 0.5518 | 0.0833 | 0.0142 | 0.3002 | 0.2712 | 0.1873 | 0.0008 | 0.1300 |
| Mn (t_1_) | 0.0591 | 0.7730 | 0.4091 | 0.1761 | 0.0005 | 0.2578 | 0.1293 | 0.5804 | 0.9219 | 0.1625 | 0.0547 | 0.1986 |
| Mn (t_2_) | 0.6677 | 0.2864 | 0.9522 | 0.7518 | 0.5323 | 0.6218 | 0.8362 | 0.3736 | 0.1408 | 0.7875 | 0.7407 | 0.8341 |
| **PS119_15-2** |  |  |  |  |  |  |  |  |  |  |  |  |
| Mn (t_0_) | 0.0975 | 0.1913 | 0.0182 | 0.9683 | 0.9021 | 0.0009 | 0.5970 | 0.2351 | 0.2246 | 0.8105 | 0.0725 | 0.6017 |
| Mn (t_1_) | 0.2043 | 0.3048 | 0.0214 | 0.9973 | 0.9998 | 0.9976 | 0.9722 | 0.9947 | 0.0184 | 0.9942 | 0.9989 | 0.8775 |
| Mn (t_2_) | 0.0660 | 0.2760 | 0.1465 | 0.2240 | 0.3116 | 0.1865 | 0.1511 | 0.0325 | 0.1407 | 0.1615 | 0.2232 | 0.0502 |
